# Effects of quercetin and derivatives on NAMPT/Sirtuin-1 metabolic pathway in neuronal cells: an approach to mitigate chemotherapy-induced cognitive impairment

**DOI:** 10.1007/s11011-025-01567-0

**Published:** 2025-03-14

**Authors:** Jeena John, Subham Das, Anu Kunnath, Jayesh Mudgal, Krishnadas Nandakumar

**Affiliations:** 1 Department of Pharmacology, Manipal College of Pharmaceutical Sciences, Manipal Academy of Higher Education, Manipal, 576104 Karnataka India; 2 Department of Pharmaceutical Chemistry, Manipal College of Pharmaceutical Sciences, Manipal Academy of Higher Education, Manipal, 576104 Karnataka India; 3 School of Pharmaceutical Sciences, Manipal University Jaipur, Jaipur, 303007 Rajasthan India; 4 Centre for Animal Research, Ethics and Training, Manipal Academy of Higher Education, Manipal, India

**Keywords:** Chemobrain, Phytochemicals, Nicotinamide phosphoribosyl transferase, Sirtuin-1

## Abstract

*Background:* The cognitive alterations observed in individuals undergoing cancer treatments have garnered more attention recently. Chemotherapy can reduce nicotinamide adenine dinucleotide (NAD+) levels by inhibiting nicotinamide phosphoribosyl transferase (NAMPT). This reduction can make cancer cells more susceptible to oxidative damage and death and may also affect non-cancerous cells, particularly the brain cells. During chemotherapy-induced suppression, the downregulation of the NAMPT-mediated NAD+/Sirtuin 1 (SIRT1) pathway may cause dyscognition. *Objective:* This study aimed to assess the role of quercetin and analogues in chemobrain and the associated mechanisms. *Methods: *The potential of quercetin and its derivatives interaction with NAMPT and SIRT1 proteins was performed using computational studies followed by their *in vitro* evaluation in SH-SY5Y cells. Molecular docking and simulation studies of human SIRT1 and NAMPT proteins with quercetin and its derivatives were performed. Differentiated SH-SY5Y cell lines were treated with quercetin and selected derivatives against Methotrexate and 5-Fluorouracil (MF) toxicity, by subjecting to cytotoxicity assay, flow cytometry, and RT-PCR analysis. *Results:* Quercetin, Rutin, and Isoquercetin showed interactions necessary in the activation process of both proteins. Cytotoxicity and flow cytometric studies demonstrated that the phytochemicals shield the differentiated SH-SY5Y cells from MF toxicity. As determined by RT-PCR investigations, NAMPT and SIRT1 gene mRNA expression was higher in test drug-treated cells at quercetin (0.12, 0.6 µM), rutin, and isoquercetin (16, 80 µM) and lower in MF-treated cells. *Conclusion:* The treatment of phytochemicals alleviated CICI by targeting NAMPT and SIRT1 proteins, which could lead to the identification of effective treatment strategies for the chemobrain.

**Graphical abstract:**

**Figure:**
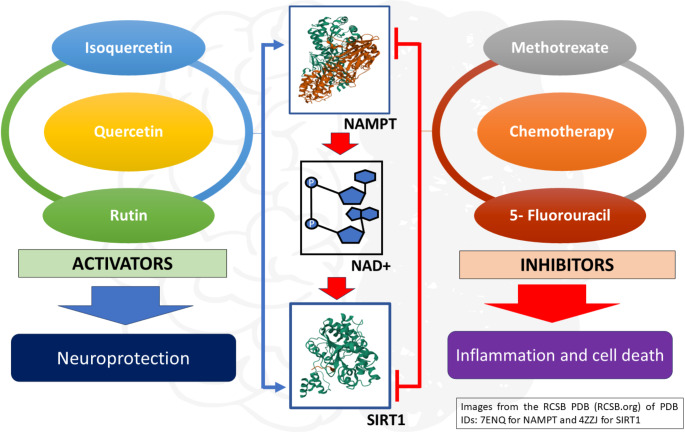
Effect of activators and inhibitors on NAMPT-mediated NAD+/SIRT1 pathway

**Supplementary Information:**

The online version contains supplementary material available at 10.1007/s11011-025-01567-0.

## Introduction

The development of chemotherapeutic agents/regimens has resulted in a considerable decrease in recurrence and a higher survival rate in several cancers, particularly breast cancer. As more individuals survive cancer treatment, they develop problems with attention, processing information, and memory, which can last months or even years following the cancer treatment. Chemotherapy-induced cognitive impairment (CICI), commonly referred to as chemobrain, describes cognitive issues in cancer patients and survivors undergoing chemotherapy treatment (Ahles et al. 2012; Janelsins et al. 2017).

Even though various pharmacological mechanisms like oxidative stress, impaired neurogenesis, and neuroinflammation have been postulated to explain chemobrain, the specific biological processes underlying the disease remain unknown. Since patients are more frequently treated with a combination of medications instead of just one chemotherapeutic drug, it is essential to take into account the molecular mechanisms of each drug, as well as their on-target and off-target effects, and the synergistic interactions between the drugs (John et al. 2021).

According to the accelerated ageing hypothesis, compared to the non-cancer population, chemotherapy results in early cognitive decline in cancer patients (Cupit-Link et al. 2017; Chang et al. 2018). Recently, the correlation of cancer, chemotherapy, ageing, and involvement of nicotinamide adenine dinucleotide (NAD+) metabolism are paving possible pathways to unravel changes at the molecular level due to chemobrain (Yaku et al. 2018; Hill et al. 2019; Kincaid and Berger 2020). A necessary coenzyme, NAD + facilitates redox processes and regulates several signalling and transcriptional events in metabolic pathways, including the tricarboxylic acid cycle, fatty acid oxidation, and glycolysis. In mammalian cells, Nicotinamide phosphoribosyl transferase, or NAMPT, serves as the rate-limiting enzyme for the metabolic reaction that produces NAD + through the salvage pathway in mammalian cells. Sirtuin-1 (SIRT1) utilises NAD + as a substrate for downstream signalling processes. Aerobic glycolysis in cancer cells upregulates energy metabolic pathways utilising nucleotides, amino acids, and lipids through increased consumption of NAD + which results in tumor proliferation and progression (Kennedy et al. 2016). NAMPT and SIRT1 are overexpressed in several malignant tumor types, including colorectal, ovarian, breast, gastric, thyroid, and prostate cancers, as well as numerous malignant lymphomas, due to the increased requirement for energy metabolism (Wang et al. 2011; Bi et al. 2011; Huang et al. 2013; Venkateshaiah et al. 2013; Maldi et al. 2013; Shackelford et al. 2013; Zhou et al. 2018). So, NAMPT inhibition, with the subsequent decline of NAD + levels, causes sensitisation of cancer cells to oxidative damage and initiation of cell death. Suppressing NAMPT-mediated NAD + synthesis by chemotherapeutic agents in cancer cells may also suppress non-cancerous cells, especially brain tissues. Deletion of NAMPT in projection neurons was found to result in neurodegeneration in mice (Wang et al. 2017). In general, overexpression of the NAMPT/SIRT1 mediated NAD + pathway in cancer and its subsequent suppression by chemotherapy may result in accelerated aging and cognitive deficits. Thus, NAMPT modulation could be vital to combat chemotherapy-induced cognitive decline. Despite the numerous benefits offered by chemotherapeutics, cancer survivors deserve long-lasting mitigation of long-term side effects caused by these agents. So, challenges in unravelling the mechanistic pathways in chemobrain and the neuroprotective role of NAMPT and SIRT1 make them suitable targets for chemobrain through the modulation of NAD + levels.

Nowadays, synthetic drugs used for treating various neurodegenerative diseases are employed to provide symptomatic relief to cancer survivors (Nguyen and Ehrlich 2020). However, coming up with pharmacological therapy for improving memory performances in CICI, which is not even established adequately for other neurodegenerative conditions, is a significant challenge. Hence, focusing on phytochemicals to reduce the disease burden would be better than concentrating on toxic chemical entities. Some phytochemicals have already been found to improve cognitive deficits in CICI (Raghu et al. 2021; John et al. 2022). Ethnopharmacological studies have documented potential medications from plant sources where it is proposed that the active compounds isolated from plants conceivably postpone neurodegeneration and improve cognitive function through their anti-inflammatory and antioxidant activities. Quercetin is a naturally available phytochemical and an important flavonoid in our diet (Costa et al. 2016; Bakoyiannis et al. 2019). Therefore, quercetin could be an ideal therapeutic strategy for CICI testing because of its anti-inflammatory and antioxidant qualities, which have been demonstrated in preclinical research. However, heterogeneity in the pathophysiological states underlying the chemo brain without compromising the anticancer effect of chemotherapeutic agents makes it difficult to focus on a targeted approach to modify the disease condition.

Recently, in our laboratory, quercetin prevented doxorubicin-induced episodic and spatial memory deficits in mammary cancer animals. It also prevented doxorubicin-induced inhibition of neurite establishment and apoptosis in rat pheochromocytoma-derived neuronal cell lines (Ramalingayya et al. 2022). Quercetin has been shown to increase the levels of NAD + and enhance the expression of SIRT1. It has also been found to activate the expression of NAMPT and AMP-activated kinase, making it an indirect activator of SIRT1 (Zhang et al. 2021; Ho et al. 2022; Jin et al. 2023). Thus, the link between preventing memory deficit and the upregulation of NAD + might be via the NAMPT pathway. Accepting the existence of heterogeneity in the pathophysiological states and focusing on cellular and molecular mechanisms with a more targeted approach can be aimed to directly modify chemobrain by reducing the toxicity of chemotherapeutic agents. Regardless of the adverse effects of cancer treatments, it is crucial to highlight the importance and numerous benefits that these therapies offer to cancer patients. Simultaneously, cancer survivors should have improved monitoring and sustained relief from the long-term side effects of chemotherapy drugs. This study focused on understanding different mechanisms underlying chemobrain, mainly the NAMPT/SIRT1 pathway, in modulating the disease condition through in silico and in vitro approaches.

Therefore, current research work aimed at evaluating the role of quercetin and its different derivatives in activating NAMPT and SIRT1 proteins, with their neuroprotective potential and may be considered essential candidates in the field of chemobrain research that could aid in underpinning the possible mechanistic involvement of NAMPT mediated NAD+/SIRT1 metabolic pathway in CICI. Considering the importance of NAMPT signalling in cancer and the possible off-target effects due to inhibition of NAMPT, particularly in the brain, our in silico studies attempt to understand the possible interactions with the NAMPT and SIRT1 proteins by quercetin and some of its analogues having neuroprotective potential but not tested against the NAMPT mediated NAD+/SIRT1 pathway using Schrodinger Maestro software. This study is the first of its kind to analyse the in-silico aspects of NAMPT and SIRT1 proteins in chemobrain, especially the computational insights into the activation of NAMPT have never been studied before in detail even though extensive research has been done for evaluating the anticancer potential of the protein. In addition, though research has been conducted to investigate the neuroprotective properties of quercetin and its derivatives, little attention has been given to comprehending the impact of methotrexate and 5-fluorouracil combinations on neuronal cells. The proposed study assessed the effects of suitable quercetin analogues in enhancing memory performance impaired due to chemotherapy, with a better understanding of underlying mechanisms through the NAMPT-mediated pathway.

## Materials and methods

### Estimation of in silico NAMPT and SIRT1 interaction of quercetin and its derivatives

Computational modeling studies were conducted using Maestro by Schrodinger (version 12.1) to analyse the effects of quercetin and selected derivatives on human SIRT1 and NAMPT proteins.

#### Selection and preparation of NAMPT and SIRT1 proteins

Studies have reported the evaluation of different molecules in several activator-bound SIRT1 complexes extracted from the protein data bank (Hubbard et al. 2013; Bakhtiari et al. 2018; Manjula et al. 2021). Here, for SIRT1, to understand the binding pattern with quercetin and its derivatives, a 3D crystal structure of the PDB ID: 4ZZJ protein was obtained from the RCSB protein database. 4ZZJ is a complex of the SIRT1 protein bound to two compounds: a small molecule sirtuin-activating compound (4TQ) and a non-hydrolysable NAD + analogue called carbaNAD. The protein was imported, reviewed, modified, and minimised using the Protein Preparation Wizard tool. During preprocessing, the structure was completed by filling in missing loops and side chains at a pH of 7 ± 1 with the addition of hydrogen atoms and the removal of water molecules. Finally, the low energy state of protein was generated by subjecting it to energy minimisation (RMSD: 0.3Å), which was then employed for simulation studies (Dai et al. 2015).

Similarly, to understand the interaction of ligands with the NAMPT protein, a crystal structure of the PDB ID: 7ENQ protein was obtained from the RCSB protein database. 7ENQ is a complex of human NAMPT protein bound to an activator molecule (NAT), which was subjected to a similar protein preparation procedure as that of SIRT1 protein at a pH of 7 ± 2 (Yao et al. 2022).

#### Ligand preparation

Here in this study, quercetin and its few naturally occurring derivatives like Rutin, Hyperoside, Isorhamnetin, Miquelianin, Catechin, Luteolin, Myricetin, Apigenin, Isoquercetin, Luteolin, Morin, and Taxifolin having neuroprotective actions were randomly selected as the ligands of interest. Quercetin was selected based on preliminary in vitro and in vivo studies performed in our laboratory. The Lig Prep tool performed ligand preparation with a force field of OPLS3e at pH 7 ± 1 for SIRT1 protein and pH of 7 ± 2 for NAMPT protein to generate possible ionisation states.

#### Molecular docking

We employed the molecular docking process to gain a deeper comprehension of how the protein and the ligand interact. This method involved creating a receptor grid to pinpoint the exact location where the ligand binds with the protein that has been minimised. It selects the atoms along the binding site of the co-ligand present in the protein, followed by the molecular docking process to obtain docking scores. These scores indicate the strength of the binding affinity between the ligand and the protein. So, once the system generates a receptor grid, the selected region in the receptor is utilised for molecular docking and other subsequent processing steps. Here, the glide tool of Schrodinger Maestro software was employed to perform molecular docking studies in extra precision (XP) mode.

#### MM-GBSA (Molecular mechanics with generalised born and surface area solvation)

The binding free energy of the ligands with the targeted proteins (NAMPT & SIRT1) was calculated using Prime MM-GBSA of the Maestro software. Here, a more negative value (kcal/mol) indicates the better strength of the complex structure(Genheden and Ryde 2015).

#### Molecular Dynamics (MD)

The ligand-protein complex is subjected to MD simulation to understand the interaction of the atoms and molecules in the biophysical system. MD simulation is based on Newtonian physics to reduce computational complexity and simulate atomic motions in physiological conditions. Here, the protein-ligand complex was subjected to MD simulation using the molecular dynamics module of Maestro software. Using the system builder, the docked protein-ligand complex was initially made under a predefined simple point charge solvent system using orthorhombic boundary conditions. After the system model was created, energy minimisation was carried out at a temperature of 300 K and a pressure of 1 bar. This process continued till a gradient threshold of 25 kcal/mol/Å was reached. The minimised complex was subjected to a 20 ns Molecular Dynamics (MD) simulation, which generated simulation interaction diagrams.

### In vitro studies

#### Procurement and maintenance of cell line

The National Centre for Cell Science (NCCS) in Pune, India, provided the human neuroblastoma SH-SY5Y cell lines. The culture medium used for these cells was Dulbecco’s Modified Eagle Medium, supplemented with 10% fetal bovine serum and 1% antibiotic-antimycotic (Gibco, Life Technologies). The cells were kept at 37 °C in an environment with 5% CO_2_.

#### Chemicals

In these studies, we used quercetin, Rutin (Sisco Research Laboratories, India), Isoquercetin (Nanjing NutriHerb BioTech, China), Retinoic acid (Sigma Aldrich, MO, USA), Acridine Orange/Ethidium bromide (HiMedia Laboratories, India), methotrexate (Biotrexate), and 5-fluorouracil (Fluracil).

#### Sulforhodamine-B (SRB) assay

After in silico drug screening, two quercetin derivatives (Rutin and Isoquercetin) were selected for further assessment of these compounds in in vitro cell culture to determine their protective effects against Methotrexate (M) and 5-Fluorouracil (F)-induced neurotoxicity in SH-SY5Y cells. SRB assay is generally performed to find the cytotoxic potential of a particular compound against a specific cell line. Here, undifferentiated SH-SY5Y cells were seeded at 3 × 10^4^ cells/well density into 96-well plates. Following the attachment of cells after 24 h, cells were treated for 48 h with different concentrations of drugs: Methotrexate (5000 µM-0.004768 µM), 5-Fluorouracil (100 µM −0.0976 µM), Quercetin (1000 µM-1.951 µM), Rutin (4000 µM-31.25 µM), Isoquercetin (4000 µM-31.25 µM). After 48 h of drug treatment, an SRB assay was performed following the procedure by Vichai and Kirtikara, (Vichai and Kirtikara 2006), where the cells were fixed with trichloroacetic acid and stained with SRB dye, followed by washing off the excess dye with acetic acid and dissolving the protein-bound dye in tris base (10 mM), and the optical density was determined at 540 nm using a microplate reader (Elx 800; BioTek instruments, USA).

#### Differentiation of SH-SY5Y cell lines and measurement of neurite length

To obtain cells having a neuron-like state, undifferentiated SH-SY5Y neuroblastoma cells were differentiated by treating with 10 µM retinoic acid (Sigma R2625) continuously for six days. After differentiation, cells were treated with different drug concentrations of quercetin (15, 3, 0.6, 0.12, 0.024, 0.0048, 0.00096 µM), rutin (2000, 400, 80, 16, 3.2, 0.64, 0.128 µM), and isoquercetin (2000, 400, 80, 16, 3.2, 0.64, 0.128 µM) for two hours before adding the toxicants [Methotrexate and 5-Fluorouracil (MF)] to determine the protective effect of the quercetin and its analogues in the presence of the toxicants. Following 48 h of drug treatment, cells were observed under an inverted microscope (Eclipse TS100F; Nikon Instruments Inc., USA), and images were captured by observing each treatment well corresponding to the drug treatments at different concentrations to assess the neurite length. Neurite length assessment was done by using the NeuronJ plugin from the ImageJ package provided by Fiji, where the step-by-step procedure for measuring the length of each neurite using ImageJ software was previously described (Pemberton et al. 2018). Differentiated cells were also further subjected to SRB assay to determine the cell viability at different doses in differentiated conditions.

#### Acridine Orange/Ethidium bromide (AO/EB) staining

Apoptosis-associated changes in the cells were detected through the use of AO/EB staining. Drug solutions were prepared in concentrations that showed increased neurite length. Hence, quercetin (0.6 µM, 0.12 µM), rutin (80 µM, 16 µM), and isoquercetin (80 µM, 16 µM) were selected and treated for two h before the addition of MF. After 48 h, cells were subjected to 50 µg/ml of each AO and EB prepared in sterile PBS (Phosphate buffered saline). After 5 min of incubation, the morphology of cells in each treatment well was examined under an inverted fluorescent microscope provided with fluorescent filters. The study of live and apoptotic cells followed the previous staining pattern described by Ribble et al. 2005; where the live cells were represented as normal green nuclei and apoptotic cells with orange nuclei. The apoptotic cells were counted in a minimum of 100 total cells to determine the percentage of apoptotic cells in each drug concentration (Ribble et al. 2005).

#### Annexin V- FITC vs. Propidium Iodide (PI) apoptosis assay

Annexin V-Fluorescein isothiocyanate staining was used to quantitatively determine apoptosis via flow cytometer (BD Accuri C6; BD Biosciences), following the manufacturer’s instructions (BD Biosciences:556547). In brief, after 48 h of drug treatment, the cells were washed with cold PBS and then resuspended in the Annexin V binding buffer. 100 µl of the cell suspension was taken, and 5 µl of Annexin V and 5 µl of propidium iodide were added, which was then incubated for 15 min at room temperature in the dark. Finally, 400 µl of binding buffer was added, and the sample was analysed using the flow cytometer (Keni et al. 2023).

#### RNA isolation, cDNA synthesis, real-time polymerase chain reaction

The RNA extraction process involved using RNAiso Plus reagent to isolate the total RNA (Takara). This was followed by adding chloroform, which was then centrifuged to separate the solution into three layers. The colourless upper aqueous phase solution containing RNA was pipetted and added with isopropanol to precipitate RNA. The pellet was resuspended in ethanol, washed twice, and dissolved in RNAase-free water. cDNA was synthesised from 1 µg of RNA using a Prime RT reagent kit (DSS Takara) per the manufacturer’s instructions in the Bio-Rad T100 thermal cycler. The qPCR reaction was performed using the TB Green Premix Ex Taq II kit (DSS Takara) in the Bio-Rad CFX96 RT-PCR detection system. The change in fold expression was determined using the 2^− ΔΔct^ method (Livak and Schmittgen 2001). The list of primers utilised can be found in Table 1.

**Table 1:** List of primer sequences used to understand the target gene expression in RT-PCR

Target	Optical density unit	Tm	Length	Sequence
NAMPT	9.2	57	22	Forward: AGGGTTACAAGTTGCTGCCACC
	9.3	57	22	Reverse: CTCCACCAGAACCGAAGGCAAT
SIRT1	9.6	57	22	Forward: TAGACACGCTGGAACAGGTTGC
	7.7	57	22	Reverse: CTCCTCGTACAGCTTCACAGTC
ACTIN	8.6	57	22	Forward: CACCATTGGCAATGAGCGGTTC
	8.3	57	22	Reverse: AGGTCTTTGCGGATGTCCACGT

### Statistical analysis

Data were expressed as mean ± SEM. GraphPad Prism version 8.4.3 (GraphPad Software, CA, USA) was used for data analysis. IC_50_ was determined by log (inhibitor) vs. response using non-linear regression analysis. One-way ANOVA followed by Dunnet’s post-hoc test was used to interpret all the other parameters in the in vitro study, and data are represented as mean ± SEM of three independent experiments. Image processing and measurement of neurite length were done with Image J software using the NeuronJ plugin (NIH, USA). A statistically significant difference was considered when *p* < 0.05 at a 95% confidence interval.

## Results

### Sirtuin-1

#### Ligand docking

Quercetin and its derivatives like Rutin, Hyperoside, Isorhamnetin, Miquelianin, Luteolin, Myricetin, Apigenin, Isoquercetin, Luteolin, Morin, and Taxifolin were docked into 4ZZJ at the allosteric region to determine the docking score. The co-crystalised ligand 4TQ had a docking score of −0.760 (Kcal/mol). The twelve selected compounds had docking scores ranging from − 5.980 to −1.196 (Kcal/mol).

The molecule 4TQ was repositioned into the crystal of the macromolecule with PDB identification number 4ZZJ using the process of redocking. The binding sites for 4TQ with 4ZZJ were at residues GLU230 as hydrogen bonding, hydrophobic (LEU206, LEU215, PRO211, PRO212, PRO230, ILE223, ILE227) residues and hydrophilic (THR209, SER229, ASN226) residues. All the selected ligands showed similar or better interactions than the co-crystallised ligand. The co-ligand and selected ligands commonly exhibited the negative charge interaction with GLU230 and the polar interaction with ASN226. Additionally, every ligand in this investigation displayed hydrophobic interactions with ILE227 and ILE223. All of the chosen ligands, except for isorhamnetin and myricetin, interacted with GLU230 through in silico docking experiments, forming a hydrogen bond with the -OH group in the flavone ring. However, isorhamnetin exhibited hydrophobic interaction with negatively charged GLU230. GLU230 is considered to be important for SIRT1 activation. Besides GLU230, all selected ligands except the co-crystallised ligand showed hydrogen bond interaction with ASN226. Quercetin showed hydrophobic interaction with ILE227 and ILE223, polar interaction with THR219, ASN226, and GLN222, and two hydrogen bond interactions with GLU230. Myricetin also exhibited hydrogen bond interactions with GLN222 and THR219. Supplementary Table 1 provides the 2D interaction diagrams and an overview of all interactions.

#### Free ligand binding energy calculations

The prime MM-GBSA was employed to calculate the binding energy of twelve selected ligands. All the ligands exhibited stability in the docked pose with ΔG binding energy < −20 kcal/mol, with the exception of rutin, morin, and taxifolin. The co-crystallised ligand’s ΔG binding energy was − 45 kcal/mol. Except for rutin, morin, and taxifolin, the co-crystallised ligand and the ligands were stable in the docked position (Table 2).

**Table 2:** Docking score, glide score, and prime MM-GBSA score of ligand-SIRT1 protein complexes

Ligand	Docking score (KCal/mol)	Glide score	MMGBSA ΔG bind score (KCal/mol)
Co-ligand (4TQ)	−0.761	−0.784	−45.74
Quercetin	−2.653	−2.653	−26.43
Rutin	−5.980	−6.008	−13.21
Hyperoside	−3.900	−3.929	−23.47
Isorhamnetin	−2.675	−2.675	−22.74
Miquelianin	−2.399	−2.428	−26.63
Catechin	−3.667	−3.667	−33.48
Isoquercetin	−3.496	−3.525	−30.47
Luteolin	−2.699	−2.739	−24.20
Myricetin	−2.325	−2.325	−22.37
Morin	−2.019	−2.247	−18.28
Apigenin	−1.196	−1.236	−24.55
Taxifolin	−1.502	−1.567	−19.56

#### Molecular dynamics

Molecular dynamics simulation experiments were carried out to obtain insight into the non-bonding interactions and binding stability of the top four drugs containing critical amino acids in the drug-binding pocket of the SIRT1 protein in a dynamic state.

For quercetin, the RMSD values for the protein and the ligand were within the acceptable limits (1–3Å) throughout the 20ns time frame. A drift in the protein-ligand complex was also observed till 7.5nsbut was found to be stable thereafter, indicating stable binding of the ligand with protein. The significant hydrogen bond interaction of GLU230, which is required for SIRT1 activation, was observed from the beginning till five ns and was not strong enough till 20 ns. Similarly, ASN226, and ILE227 interactions were also maintained throughout the simulation time with varying intensities (Supplementary Fig. 1).

For isoquercetin, ligand and protein drift from the reference trajectory was observed for the initial 7.5 ns. But later, stable protein and ligand were observed for the remaining simulation time within the acceptable range of RMSD. Desired interactions like GLU230, ASN226, and ILE227 were also maintained throughout the simulation time (Supplementary Fig. 2).

For rutin, ligand and protein drift from the reference trajectory was observed for the initial five ns. But later, stable protein and ligand were observed for the remaining simulation time within the acceptable range of RMSD. Important interactions like GLU230, ILE227, and ASN226 were maintained throughout the simulation (Supplementary Fig. 3).

For miquelianin, ligand and protein drift from the reference trajectory was observed for the initial 10 ns. But later, stable protein and ligand were observed for the remaining simulation time within the acceptable range of RMSD. Interactions like GLU230 and ASN226 were maintained firmly for the initial 11ns but were observed to be diminished for the later simulation time (Supplementary Fig. 4).

For hyperoside, the ligand and the protein showed significant drift in the RMSD values, signifying the unstable ligand-protein complex. However, desired interactions like GLU230, ASN226, and ILE223 were maintained throughout the simulation (Supplementary Fig. 5).

### NAMPT

#### Ligand docking

Quercetin and its derivatives like Rutin, Hyperoside, Isorhamnetin, Miquelianin, Catechin, Luteolin, Myricetin, Apigenin, Isoquercetin, Luteolin, Morin, and Taxifolin were docked into 7ENQ at allosteric region to determine the docking score. Co-crystallised ligand NAT had a docking score of −6.488 (KCal/mol). The docking scores of the chosen twelve compounds ranged from − 11.711 to −6.280 (KCal/mol).

Re-docking of the ligand NAT into the macromolecule crystal of PDB: 7ENQ was performed. The places where NAT and 7ENQ bound were at ASP219, where a hydrogen bond was formed with the protein’s -OH group through a water molecule. This hydrogen link is also present with the side chains of residue VAL242. Furthermore, a hydrogen bond was established between NAT’s center amide nitrogen and TYR188 via a water molecule.

All selected ligands showed similar or better interactions than the co-crystallised ligand. The researchers performed structural optimisation to improve NAT’s potency and pharmacological properties by synthesising its derivatives, where they discovered that NAT-5r enhanced the enzymatic activity of NAMPT. They found that the crucial interaction necessary for boosting the NAMPT activity is LYN189, which was not found in NAT. However, insilico docking results showed that this desired interaction was observed in quercetin and its different derivatives, such as Rutin, Isoquercetin, Hyperoside, and Miquelianin. Apart from this, other common hydrogen bond interactions in all the ligands were found to be ASP219, VAL242 and SER275. Supplementary Table 2 provides the 2D interaction diagrams and an overview of all interactions.

#### Free ligand binding energy calculations

The binding energies of twelve chosen ligands were determined using the prime MM-GBSA approach in this investigation. It was discovered that every ligand had an ΔG binding energy of less than − 20 kcal/mol and was stable in the docked position. With a ΔG binding energy of −59 kcal/mol, the co-crystallised ligand quercetin was stable in the docked position with its counterparts (Table 3).

**Table 3:** Docking score, glide score, and prime MM-GBSA score NAMPT protein complexes

Ligand	Docking score (KCal/mol)	Glide score	MMGBSA ΔG bind score (KCal/mol)
Co-ligand (NAT)	−6.488	−6.489	−59.42
Quercetin	−9.194	−9.226	−42.75
Rutin	−11.711	−13.631	−58.10
Hyperoside	−10.110	−10.139	−49.58
Isorhamnetin	−7.886	−7.918	−35.35
Miquelianin	−9.681	−9.709	−38.21
Catechin	−8.514	−8.514	−40.85
Isoquercetin	−10.050	−10.079	−45.56
Luteolin	−8.652	−8.692	−42.94
Myricetin	−8.557	−8.594	−35.12
Morin	−6.985	−7.240	−31.35
Apigenin	−6.280	−6.320	−35.82
Taxifolin	−8.379	−8.426	−47.50

#### Molecular dynamics

Similar to SIRT1 dynamics studies, four compounds were selected for molecular dynamics studies to simulate the actions of protein in the presence of ligands under normal physiological processes. Apart from quercetin, rutin, isoquercetin, miquelianin and hyperoside were selected for molecular dynamic studies based on the docking score, amino acid interactions as well as the stability of the ligand with the protein.

Both ligand and the protein were within the acceptable RMSD range of 1–3 Å for quercetin. The protein-ligand complex was stable throughout the simulation time except from around 13ns to 17.5ns but later was shown within the desired RMSD range. Also, the interactions like ASP219, VAL242, and SER275 were maintained, but the LYN189 amino acid interaction was feeble after 12.5ns. Interactions like HIS191, ARG311, ILE378, GLU376, and VAL350 were found additional to the interactions obtained during the docking (Supplementary Fig. 6).

For Isoquercetin, both ligand and the protein were within the acceptable RMSD range of 1–3 Å. Also, interactions like VAL242, SER275, and LYN189 were maintained, but the ASP219 amino acid interaction was found only during the initial 6ns and later diminished in the trajectory frame (Supplementary Fig. 7).

For Rutin, both the ligand and the protein were within the acceptable RMSD range of 1–3 Å. Also, interactions like ASP219, VAL242, SER275, and LYN189 were maintained during the simulation. Interactions like ILE378, VAL350, and ARG311 also showed hydrogen bond interactions via water molecules throughout the simulation (Supplementary Fig. 8).

For Miquelianin, atoms were displaced in both ligands and the protein for the initial 6ns, but was later found to fall within the acceptable RMSD range of 1–3 Å. Also, interactions like ASP219, SER275, and LYN189 were maintained only during the beginning but diminished in the later period of the simulation. Additionally, interactions like GLU376, ARG349, THR304, PRO346, VAL350, and HIS191 were found (Supplementary Fig. 9).

For Hyperoside, there was a huge change in the RMSD values of both ligand and the protein for the initial 10ns, but it was later found to fall within the acceptable RMSD range of 1–3 Å. Also, interactions like ASP219 and LYN189 were not maintained uniformly during the trajectory simulation. Apart from this, amino acid interactions like SER275, ARG311, GLU376, and ASP184 were found in addition to those obtained from docking (Supplementary Fig. 10).

### In vitro studies

#### Cytotoxicity assay

**Fig. 1:**
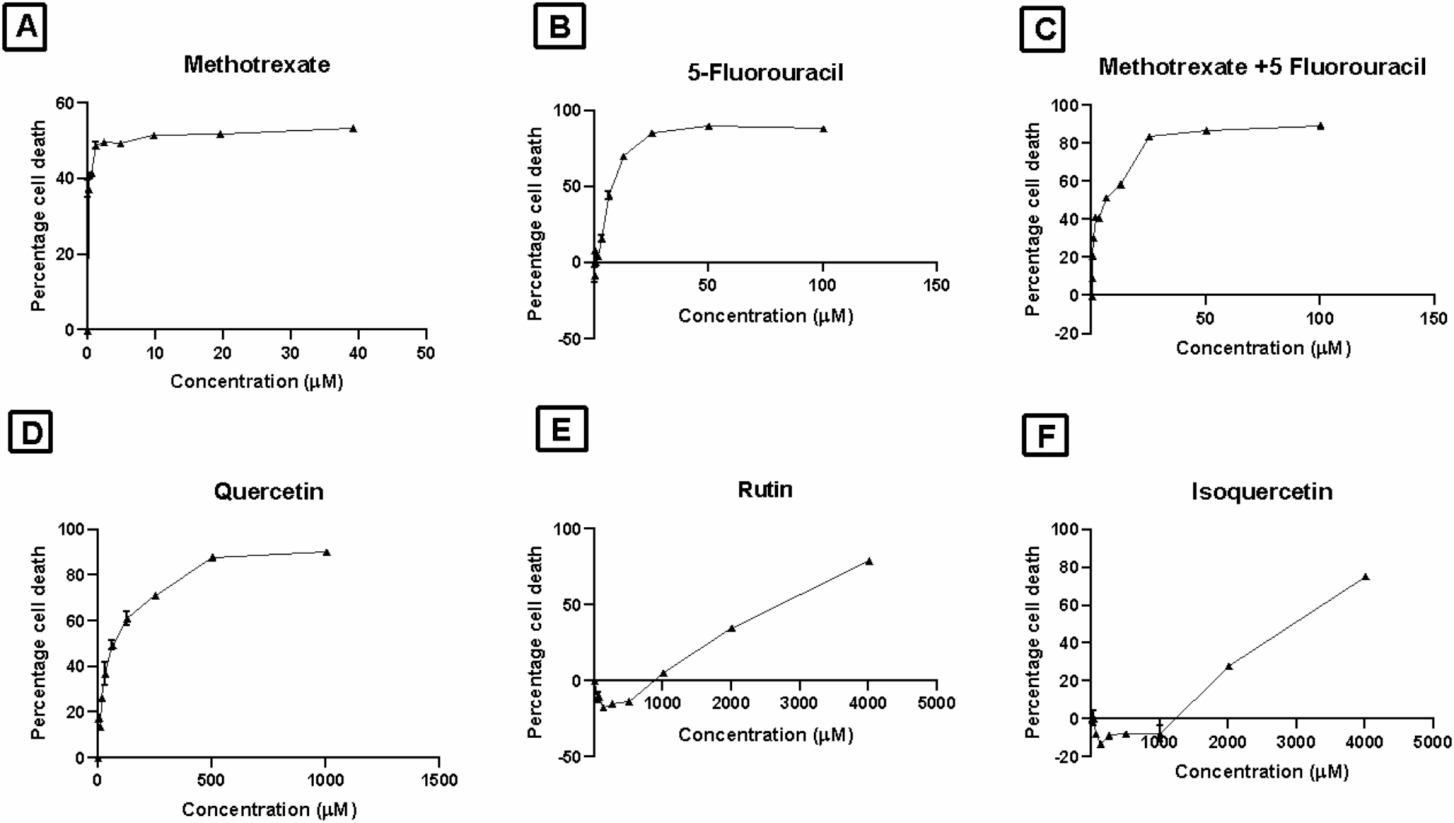
Effect of different concentrations of **A**: Methotrexate; **B**: 5-Fluorouracil; **C**: Methotrexate + 5-Fluorouracil; **D**: Quercetin; **E**: Rutin; F: Isoquercetin; on the percentage cell death of SH-SY5Y cells after 48 h of incubation. The data is represented as mean ± SEM of three independent experiments

The half-maximal inhibitory concentration (IC50) value of test drugs on undifferentiated SH-SY5Y cells was determined using the Sulphorhodamine B assay. The IC_50_ values after treatment for 48 h were 6.032 ± 0.2519 µM for methotrexate, 7.824 ± 0.4566 µM for 5-Fluorouracil, 60.07 ± 0.6083 µM for quercetin, 2504 ± 4.163 µM for rutin, and 2780 ± 0.5774 µM for isoquercetin. Toxicity was induced by the combination of equimolar concentration of methotrexate and 5-Fluorouracil, which was found to be 4.192 ± 0.03688 µM (Fig. 1; Table 4).

**Table 4:** Calculated IC_50_ values of test compounds after 48 h incubation with SH-SY5Y cells

Test compounds	Calculated IC_50_ (µM)
Methotrexate	6.032 ± 0.2519
5- Fluorouracil	7.824 ± 0.4566
Methotrexate + 5- Fluorouracil	4.192 ± 0.03688
Quercetin	60.07 ± 0.6083
Rutin	2504 ± 4.163
Isoquercetin	2780 ± 0.5774

#### Effect of quercetin and its analogues on cell viability in undifferentiated SH-SY5Y cells

**Fig. 2:**
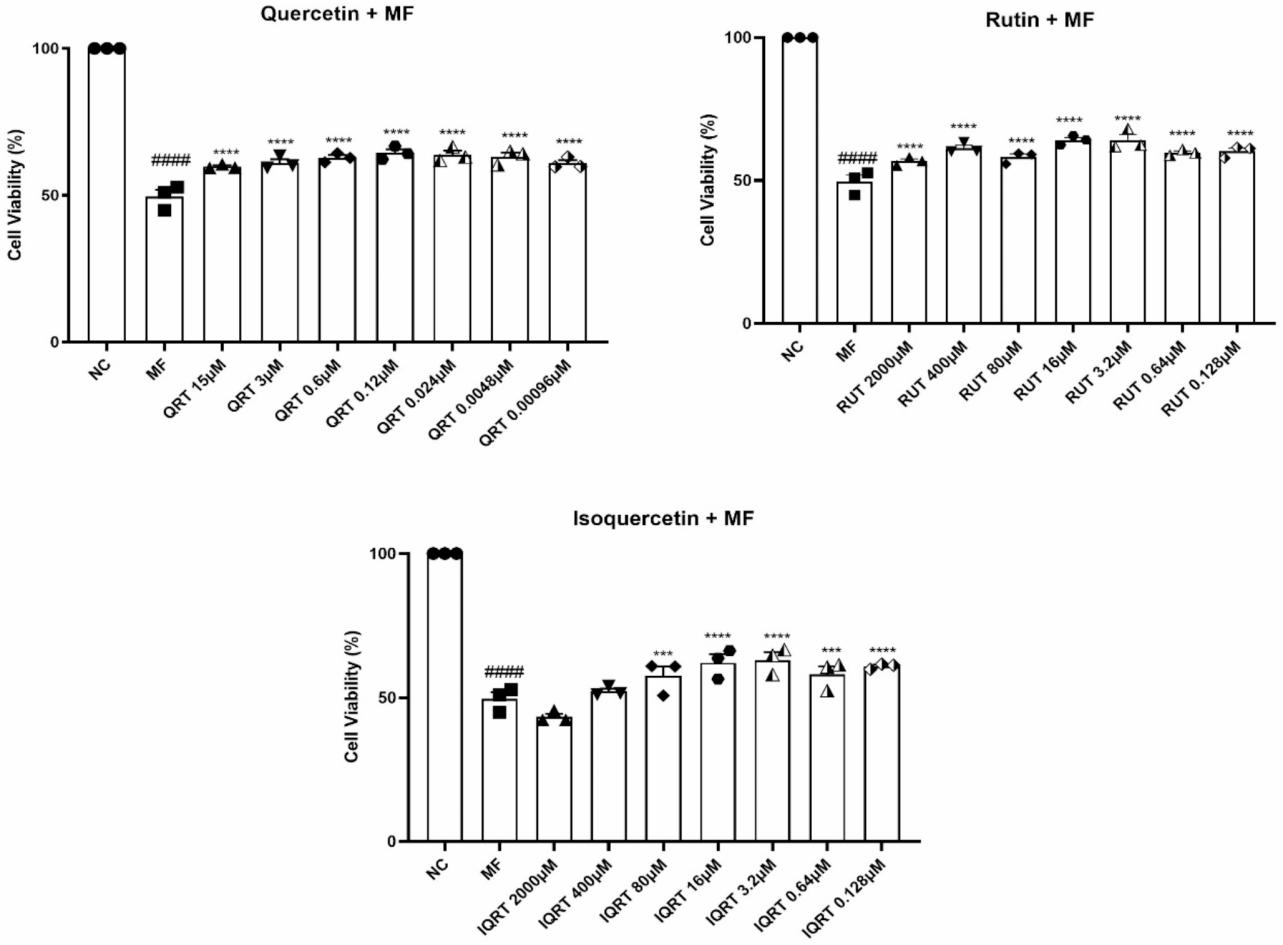
Effect of 2 h pretreatment of Quercetin, Rutin, Isoquercetin on cell viability on MF-induced toxicity in undifferentiated SH-SY5Y cells. Data represents mean ± SEM analysed by One-way ANOVA followed by Dunnet’s post-hoc test. ####*p* < 0.0001 compared to normal control. ***p < 0.001, ****p < 0.0001 compared to MF control. (QRT: Quercetin, RUT: Rutin, IQRT: Isoquercetin)

Treatment of MF (4.192 µM) resulted in 50.47% cell death in undifferentiated SH-SY5Y cells. Seven concentrations that produced more than 70% viability were selected for each drug: Quercetin (15, 3, 0.6, 0.12, 0.024, 0.0048, 0.00096 µM), Rutin (2000, 400, 80, 16, 3.2, 0.64, 0.128 µM), Isoquercetin (2000, 400, 80, 16, 3.2, 0.64, 0.128 µM) and tested for assessing their neuroprotective potential in the presence of the MF (4.192 µM) by treating the cells with the test drugs two h before the addition of the toxicants in undifferentiated SH-SY5Y cells. The absorbance of the control group is taken as 100% of the cell viability. One-way ANOVA revealed a statistically significant difference in cell viability (F_22,46_=38.30, *p* < 0.0001) between the treatment groups in undifferentiated SH-SY5Y cells. Exposure of undifferentiated SH-SY5Y cells to MF showed a significant decrease in the percentage of cell viability (49.53 ± 2.363 vs. 100%, *p* < 0.0001) compared to the untreated cells. For quercetin, all the selected drug concentrations like 15 µM (59.76 ± 0.480 vs. 49.53 ± 2.363, *p* < 0.001), 3 µM (60.96 ± 1.303 vs. 49.53 ± 2.363, *p* < 0.0001), 0.6 µM (62.76 ± 0.889 vs. 49.53 ± 2.363, *p* < 0.0001), 0.12 µM (64.39 ± 1.301 vs. 49.53 ± 2.363, *p* < 0.0001), 0.024 µM (63.79 ± 1.388 vs. 49.53 ± 2.363, *p* < 0.0001), 0.0048 µM (63.14 ± 1.429 vs. 49.53 ± 2.363, *p* < 0.0001), 0.00096 µM (60.83 ± 1.092 vs. 49.53 ± 2.363, *p* < 0.0001) prevented MF-induced toxicity. Similarly, for rutin, all the selected drug concentrations like 2000 µM (56.77 ± 0.816 vs. 49.53 ± 2.363, *p* < 0.0001), 400 µM (61.30 ± 1.049 vs. 49.53 ± 2.363, *p* < 0.0001), 80µM (58.14 ± 1.157 vs. 49.53 ± 2.363, *p* < 0.001), 16 µM (64.13 ± 0.926 vs. 49.53 ± 2.363, *p* < 0.0001), 3.2 µM (64.13 ± 2.016 vs. 49.53 ± 2.363, *p* < 0.0001), 0.64 µM (59.76 ± 0.594 vs. 49.53 ± 2.363, *p* < 0.0001), 0.128 µM (60.24 ± 1.182 vs. 49.53 ± 2.363, *p* < 0.0001) prevented MF-induced toxicity. Furthermore, pretreatment of isoquercetin also reversed toxicant insult at drug concentrations like 80 µM (57.50 ± 3.401 vs. 49.53 ± 2.363, *p* < 0.001), 16 µM (62.12 ± 2.919 vs. 49.53 ± 2.363, *p* < 0.0001), 3.2 µM (63.10 ± 2.605 vs. 49.53 ± 2.363, *p* < 0.0001), 0.64 µM (58.10 ± 2.875 vs. 49.53 ± 2.363, *p* < 0.001), 0.128 µM (60.88 ± 0.546 vs. 49.53 ± 2.363, *p* < 0.0001). Still, it did not show enhanced cell viability at 2000 µM (43.34 ± 1.112) and 400 µM (52.19 ± 0.926) (Fig. 2) (Supplementary Tables 3, 4, and 5).

#### Effect of quercetin and its analogues on cell viability in differentiated SH-SY5Y cells

**Fig. 3:**
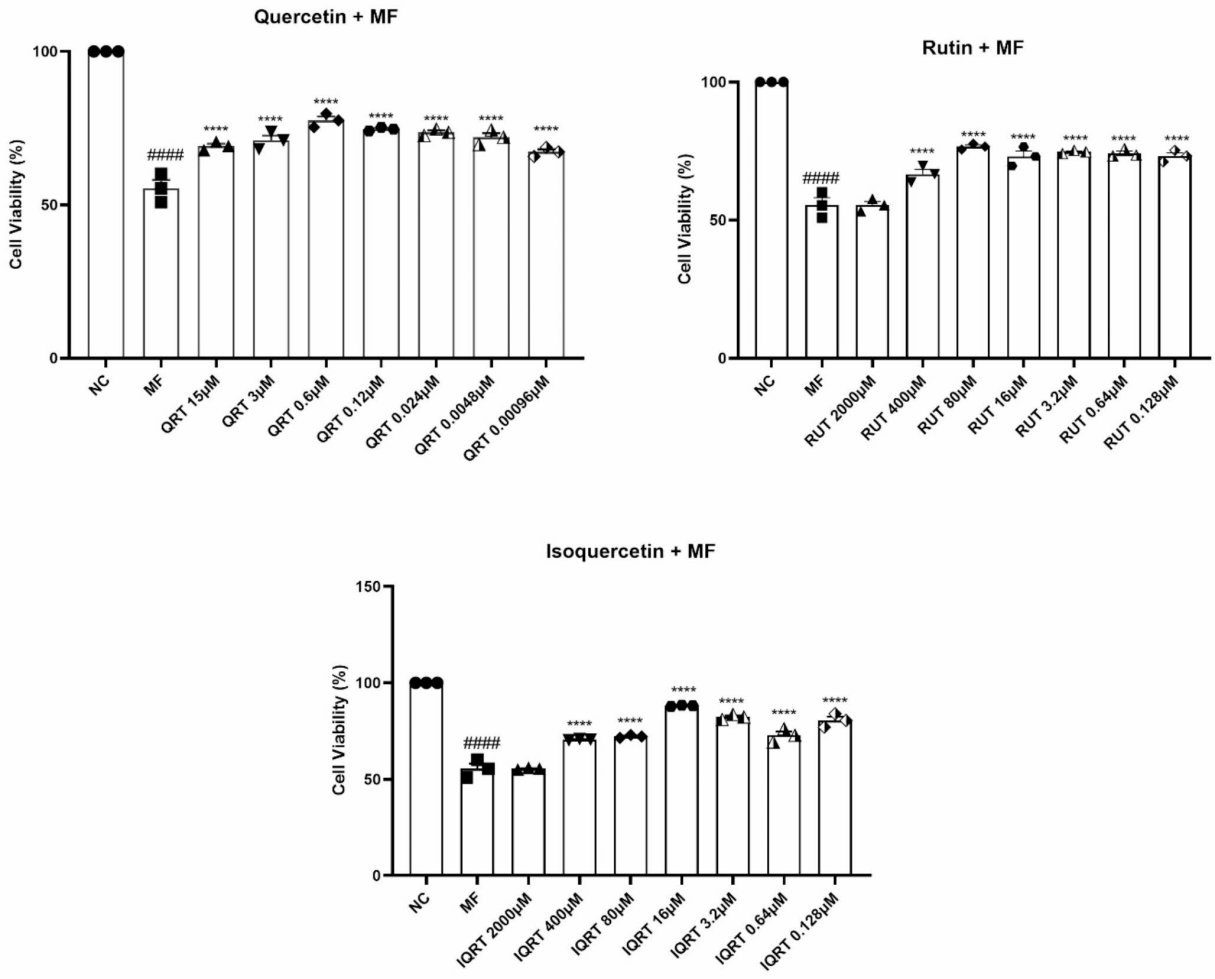
Effect of 2 h pretreatment of Quercetin, Rutin, Isoquercetin on cell viability on MF-induced toxicity in differentiated SH-SY5Y cells. Data represents mean ± SEM analysed by One-way ANOVA followed by Dunnet’s post-hoc test. ####*p* < 0.0001 compared to normal control. ****p < 0.0001 compared to MF control. (QRT: Quercetin, RUT: Rutin, IQRT: Isoquercetin)

Treatment of MF (4.192 µM) resulted in 44.54% cell death in differentiated SH-SY5Y cells. One-way ANOVA revealed that there was a statistically significant difference in cell viability (F_22,46_=64.53, *p* < 0.0001) between the treatment groups in differentiated SH-SY5Y cells. Exposure of differentiated SH-SY5Y cells to MF showed a significant decrease in the percentage of cell viability (55.46 ± 2.664 vs. 100%, *p* < 0.0001) compared to the untreated cells. For quercetin, all selected drug concentrations like 15 µM (69.20 ± 0.773 vs. 55.46 ± 2.664, *p* < 0.0001), 3 µM (70.98 ± 1.632 vs. 55.46 ± 2.664, *p* < 0.0001), 0.6 µM (77.53 ± 1.289 vs. 55.46 ± 2.664, *p* < 0.0001), 0.12 µM (74.70 ± 0.343 vs. 55.46 ± 2.664, *p* < 0.0001), 0.024 µM (73.66 ± 0.601 vs. 55.46 ± 2.664, *p* < 0.0001), 0.0048 µM (72.02 ± 1.375 vs. 55.46 ± 2.664, *p* < 0.0001), 0.00096 µM (67.26 ± 0.859 vs. 55.46 ± 2.664, *p* < 0.0001) prevented MF-induced toxicity. Similarly, pretreatment of rutin at drug concentrations like 400 µM (66.67 ± 1.718 vs. 55.46 ± 2.664, *p* < 0.0001), 80 µM (76.64 ± 0.601 vs. 55.46 ± 2.664, *p* < 0.0001), 16 µM (73.07 ± 1.976 vs. 55.46 ± 2.664, *p* < 0.0001), 3.2 µM (74.70 ± 0.343 vs. 55.46 ± 2.664, *p* < 0.0001), 0.64 µM (74.23 ± 0.839 vs. 55.46 ± 2.664, *p* < 0.0001), 0.128 µM (73.21 ± 1.203 vs. 55.46 ± 2.664, *p* < 0.0001) prevented MF-induced toxicity except at 2000 µM (55.51 ± 1.289 vs. 55.46 ± 2.664). Furthermore, pretreatment of isoquercetin at drug concentrations like 400 µM (70.54 ± 0.171 vs. 55.46 ± 2.664, *p* < 0.0001), 80 µM (86.76 ± 0.601 vs. 55.46 ± 2.664, *p* < 0.0001), 16 µM (86.61 ± 1.031 vs. 55.46 ± 2.664, *p* < 0.0001), 3.2 µM (82.29 ± 0.773 vs. 55.46 ± 2.664, *p* < 0.0001), 0.64 µM (72.77 ± 1.976 vs. 55.46 ± 2.664, *p* < 0.0001), 0.128 µM (80.51 ± 1.976 ± 2.664, *p* < 0.0001) but did not showed significant increase in cell viability at 2000 µM (55.51 ± 0.257 vs. 55.46 ± 2.664) (Fig. 3) (Supplementary Tables 3, 4, and 5).

#### Effect of quercetin and its analogues on neurite length in differentiated SH-SY5Y cells

**Fig. 4:**
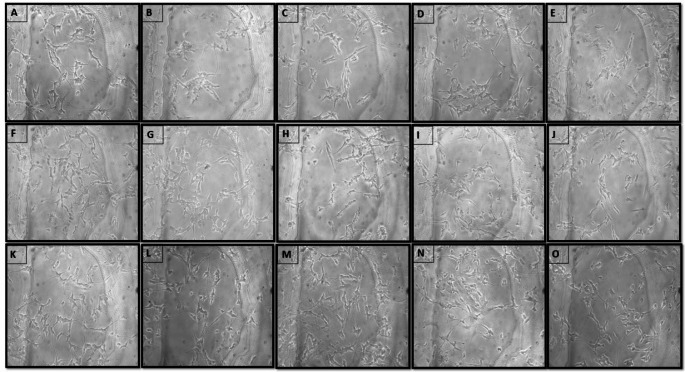
Effect of 2 h pretreatment of QRT, RUT and IQRT concentrations on the neurite length on MF-induced toxicity in differentiated SH-SY5Y cells after 48 h of incubation; **A**: Normal Control; **B**: MF Control; **C**: Quercetin (15 µM); **D**: Quercetin (3 µM); **E**: Quercetin (0.6 µM); **F**: Quercetin (0.12 µM); **G**: Quercetin (0.024 µM); **H**: Rutin (400 µM); **I**: Rutin (80 µM); **J**: Rutin (16 µM); **K**: Rutin (3.2 µM); **L**: Isoquercetin (400 µM); **M**: Isoquercetin (80 µM); **N**: Isoquercetin (16 µM); **O**: Isoquercetin (3.2 µM). Images are representations of three independent experiments

**Fig. 5:**
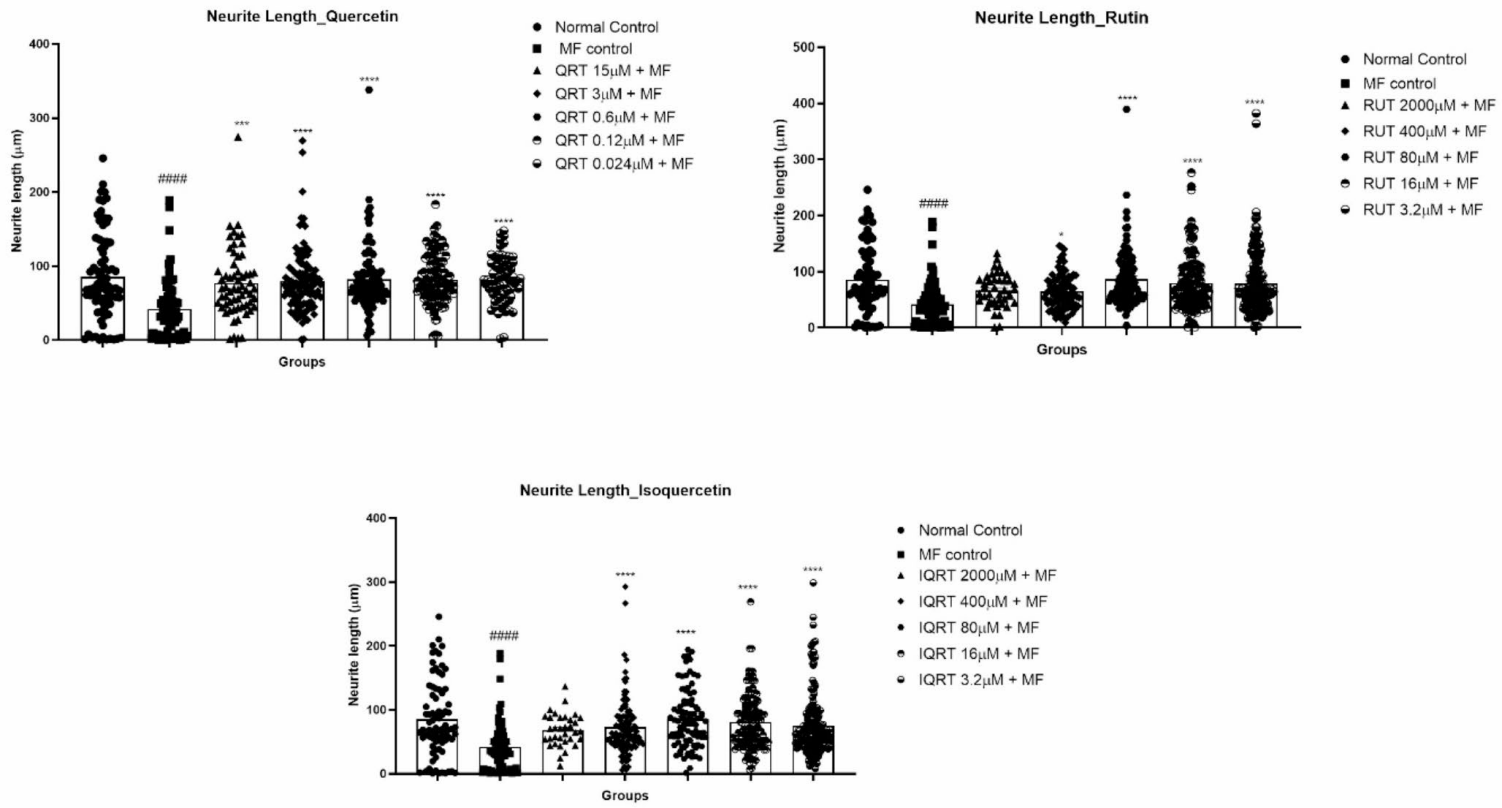
Effect of 2 h pretreatment of different test drug concentrations on the neurite length on MF-induced toxicity in differentiated SH-SY5Y cells after 48 h of incubation. Data represents mean ± SEM of three trials analysed by One-way ANOVA followed by Dunnet’s post-hoc test. ####*p* < 0.0001 compared to normal control. *p < 0.05, ****p < 0.001,****p < 0.0001 compared to MF control. (QRT: Quercetin, RUT: Rutin, IQRT: Isoquercetin)

Four to five concentrations were selected from each test drug treatment based on the cell viability for neurite length measurement. One-way ANOVA revealed a statistically significant difference in neurite length (F_16,1588_=4.256, *p* < 0.0001) between the treatment groups in differentiated SH-SY5Y cells. Treatment of differentiated SH-SY5Y cells with MF significantly reduced neurite length (41.77 ± 4.715 vs. 85.38 ± 6.717, *p* < 0.0001) compared to the untreated cells, showing the inhibitory action of MF in neurite development. For quercetin, drug concentrations like 15µM (76.74 ± 5.916 vs. 41.77 ± 4.715, *p* < 0.001), 3 µM (70.98 ± 1.632 vs. 41.77 ± 4.715, *p* < 0.0001), 0.6 µM (77.53 ± 1.289 vs. 41.77 ± 4.715, *p* < 0.0001), 0.12 µM (74.70 ± 0.343 vs. 41.77 ± 4.715, *p* < 0.0001), 0.024 µM (73.66 ± 0.601 vs. 41.77 ± 4.715, *p* < 0.0001) showed an increase in neurite length compared to the MF-treated group. Similarly, pretreatment of rutin at drug concentrations like 400 µM (64.85 ± 3.451 vs. 41.77 ± 4.715, *p* < 0.05), 80 µM (83.83 ± 5.148 vs. 41.77 ± 4.715, *p* < 0.0001), 16 µM (79.72 ± 4.537 vs. 41.77 ± 4.715, *p* < 0.0001), 3.2 µM (79.01 ± 4.500 vs. 41.77 ± 4.715, *p* < 0.0001) prevented MF-induced neurite growth inhibition. Furthermore, pretreatment of isoquercetin at drug concentrations like 400 µM (73.11 ± 4.606 vs. 41.77 ± 4.715, *p* < 0.001), 80 µM (85.35 ± 4.738 vs. 41.77 ± 4.715, *p* < 0.0001), 16 µM (81.07 ± 3.769 vs. 41.77 ± 4.715, *p* < 0.0001), and 3.2 µM (75.15 ± 3.798 vs. 41.77 ± 4.715, *p* < 0.0001) showed significant increase in neurite length compared MF-treated group (Figs. 4 and 5) (Supplementary Table 6).

#### Effect of quercetin and its analogues on MF-induced apoptosis in differentiated SH-SY5Y cells

Two drug concentrations that showed increased neurite length were selected from each drug treatment for further studies.

##### Acridine Orange/Ethidium Bromide (AO/EB) staining

**Fig. 6:**
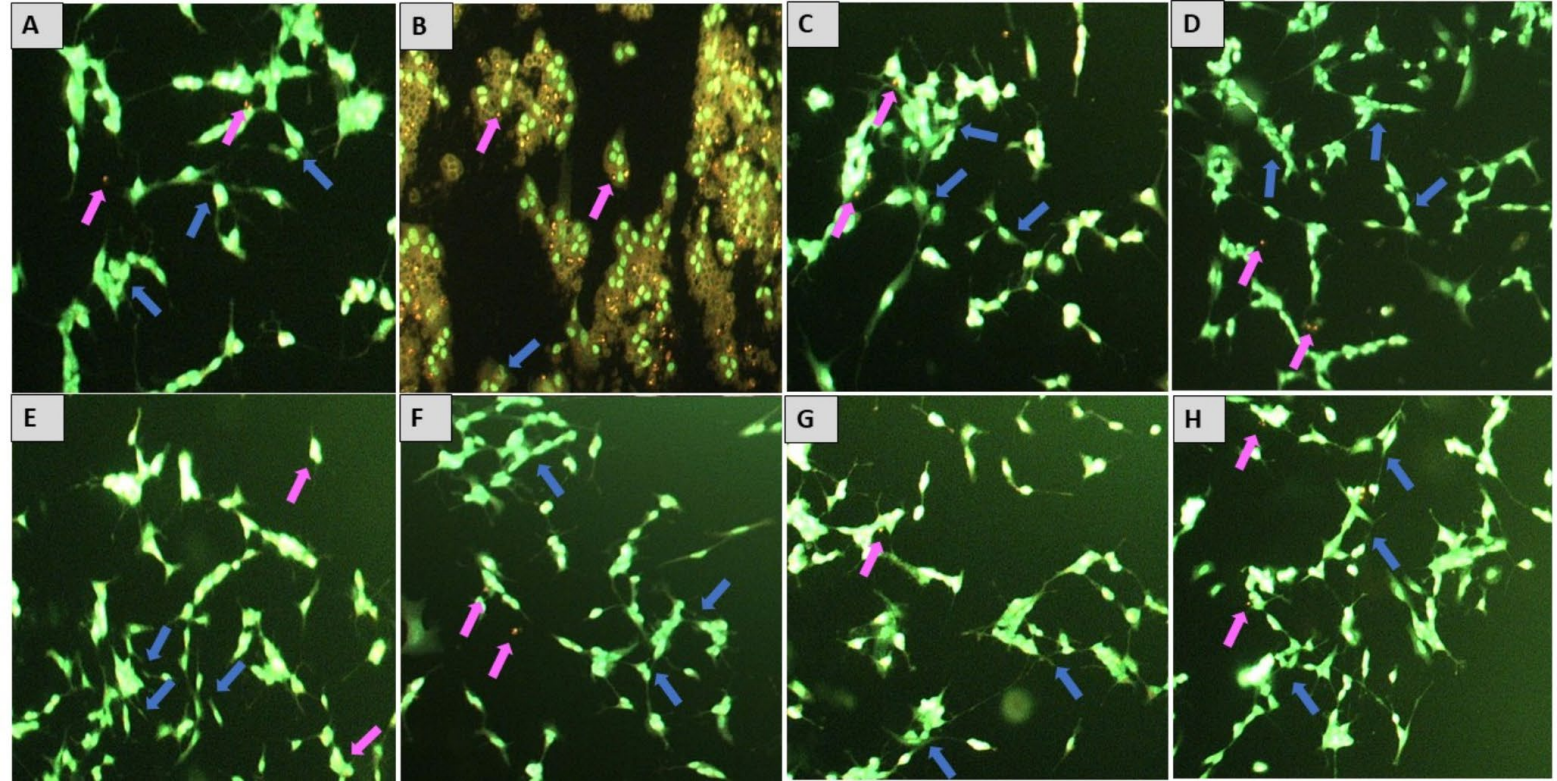
Effect of QRT, RUT and IQRT concentrations on apoptosis after AO/EB staining upon MF-induced toxicity in differentiated SH-SY5Y cells. **A**: Normal Control; **B**: MF Control; **C**: Quercetin (0.12 µM); **D**: Quercetin (0.6 µM); **E**: Rutin (16 µM); **F**: Rutin (80 µM); **G**: Isoquercetin (16 µM); **H**: Isoquercetin (80 µM); Purple arrow represents an apoptotic cell, and blue arrow represents normal cell. Images are representations of three independent experiments

**Fig. 7:**
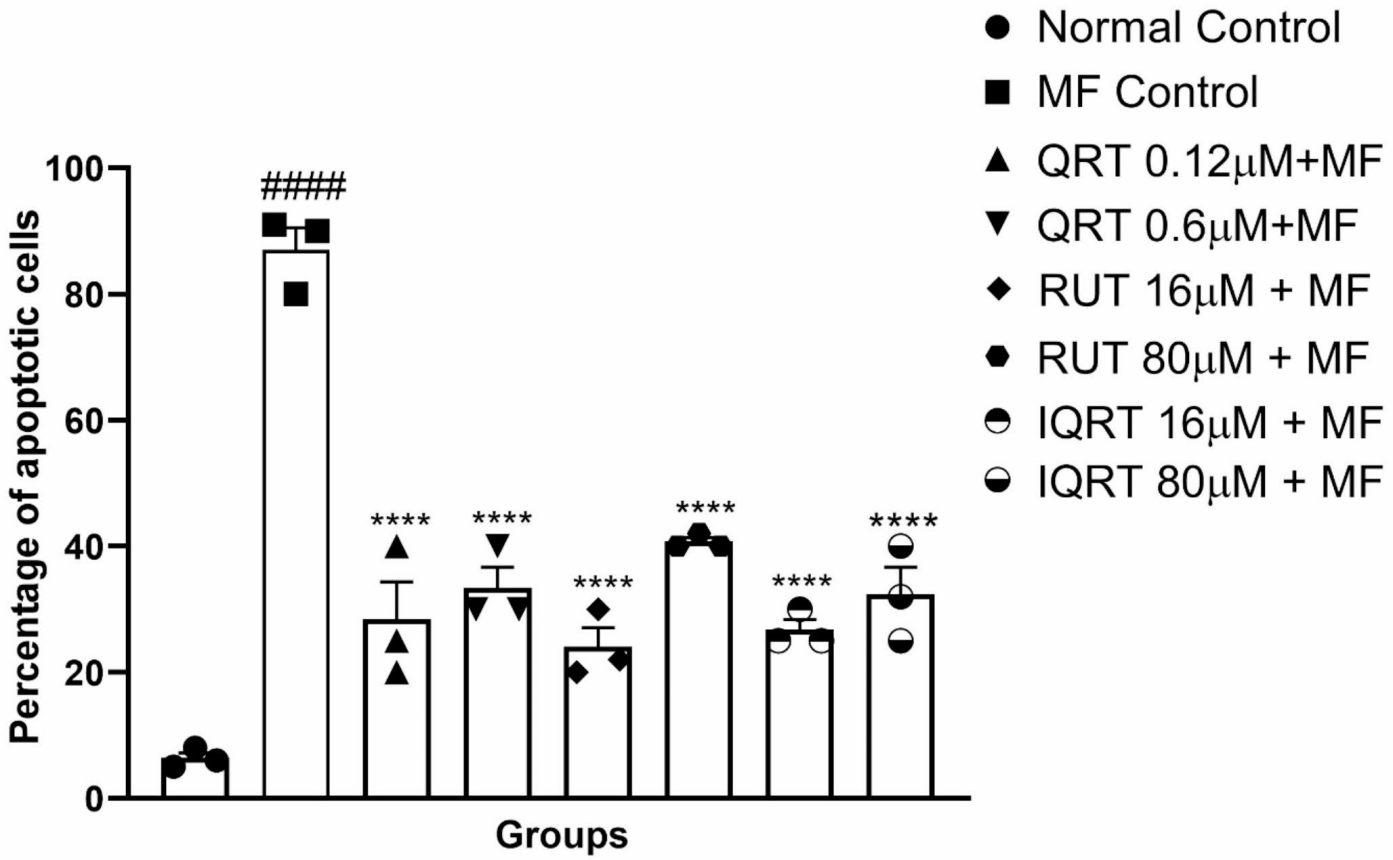
Effect of different QRT, RUT, and IQRT concentrations on percentage cell death after AO/EB staining upon MF-induced toxicity in differentiated SH-SY5Y cells. Data represents mean ± SEM of three independent experiments (*n* = 3) analysed by One-way ANOVA followed by Dunnet’s post-hoc test. ####*p* < 0.0001 compared to normal control group, ****p < 0.0001 compared to MF control group. (QRT: Quercetin; RUT: Rutin; IQRT: Isoquercetin)

One-way ANOVA revealed a statistically significant difference in the percentage of cell death (F_7,16_=47.41, *p* < 0.0001) between the treatment groups in AO/EB staining. After being treated with MF, differentiated SH-SY5Y cells stained with AO/EB showed an increased percentage of cell death (87 ± 3.512 vs. 6.333 ± 0.8819, *p* < 0.0001) compared to the normal control. This was evidenced by cells undergoing apoptosis, which had bright green to orange nuclei. However, the pretreatment with quercetin, rutin, and isoquercetin exhibited a statistically significant reduction in cell death compared to the MF control group, as indicated by the more significant number of green nuclei than the group treated with MF alone. For quercetin, drug concentrations like 0.6 µM (33.33 ± 3.33 vs. 87 ± 3.512, *p* < 0.001), 0.12 µM (28.33 ± 6.009 vs. 87 ± 3.512, *p* < 0.0001), showed a significant decrease in percentage cell death compared to the MF-treated group. For rutin, drug concentrations like 80 µM (40.67 ± 1.155 vs. 87 ± 3.512, *p* < 0.001), and 16 µM (24 ± 3.055 vs. 87 ± 3.512, *p* < 0.0001), showed significant decreases in percentage cell death compared to the MF-treated group. Similarly, for isoquercetin, drug concentrations like 80 µM (32.33 ± 4.333 vs. 87 ± 3.512, *p* < 0.001), 16 µM (26.67 ± 1.667 vs. 87 ± 3.512, *p* < 0.0001) showed a significant decrease in percentage cell death compared to the MF-treated group (Figs. 6 and 7).

##### FITC annexin V/ propidium iodide apoptosis assay

Annexin V and Propidium Iodide staining further confirmed apoptosis induction by MF using flow cytometry. During flow cytometric analysis, most cells in the treatment groups were observed to be in the live and apoptotic stages. However, there were fewer cells in the necrotic stage. One-way ANOVA revealed that there was a statistically significant difference in the live cell population (F_7,8_=15.82, *p* = 0.0004), apoptotic population (F_7,8_=14.19, *p* = 0.0006), and necrotic population (F_7,8_=6.349, *p* = 0.0092) between the treatment groups. The live cell population in the MF control group was significantly (49.31 ± 0.3600 vs. 79.24 ± 6.420, *p* < 0.001) lower than those in the normal control group. In contrast, the apoptotic (50.63 ± 0.3650 vs. 20.66 ± 6.340, *p* < 0.001) (F_7,8_=14.19, *p* = 0.0006) and necrotic population (0.09500 ± 0.005000 vs. 0.03000 ± 01000, *p* < 0.01) (F_7,8_=6.349, *p* = 0.0092) were found to be higher compared to the normal control group indicating that the MF treatment caused an increase in apoptotic and necrotic events while reducing live cell populations. However, the test drug treatment groups like quercetin at 0.12 µM (65.23 ± 0.0300 vs. 49.31 ± 0.3600, *p* < 0.01), 0.6 µM (63.99 ± 0.1000 vs. 49.31 ± 0.3600, *p* < 0.01), isoquercetin at 16 µM (65.53 ± 0.5650 vs. 49.31 ± 0.3600, *p* < 0.01), showed a significantly higher live cell population than the MF control group, except for the rutin-treated group. Likewise, a significant reduction in apoptotic population was observed in quercetin at 0.6 µM (36.10 ± 1.010 vs. 50.63 ± 0.3650, *p* < 0.05), 0.12 µM (34.65 ± 0.1450 vs. 50.63 ± 0.3650, *p* < 0.01), rutin at 16 µM (38.36 ± 1.375 vs. 50.63 ± 0.3650, *p* < 0.05), and isoquercetin at 16µM (34.38 ± 0.6650 vs. 50.63 ± 0.3650, *p* < 0.01) compared to the MF-treated group. A similar trend was also observed in the necrotic population where quercetin at 0.12 µM (0.03500 ± 0.005000 vs. 0.09500 ± 0.005000, *p* < 0.01), rutin at 16 µM (0.03500 ± 0.01500 vs. 0.09500 ± 0.005000, *p* < 0.01), 80 µM (0.05000 ± 0.01000 vs. 0.09500 ± 0.005000, *p* < 0.05) and isoquercetin at 16 µM (0.02500 ± 0.005000 vs. 0.09500 ± 0.005000, *p* < 0.01), 80 µM (0.04000 ± 0.01000 vs. 0.09500 ± 0.005000, *p* < 0.05) showed a reduction in the percentage of necrotic cell population compared to the MF-treated group. Among the treatment groups, there was a slight dose-dependent decrease in the live cell population and an increase in the apoptotic and necrotic population, similar to the AO/EB stainingFig. . 8 and 9).

**Fig. 8:**
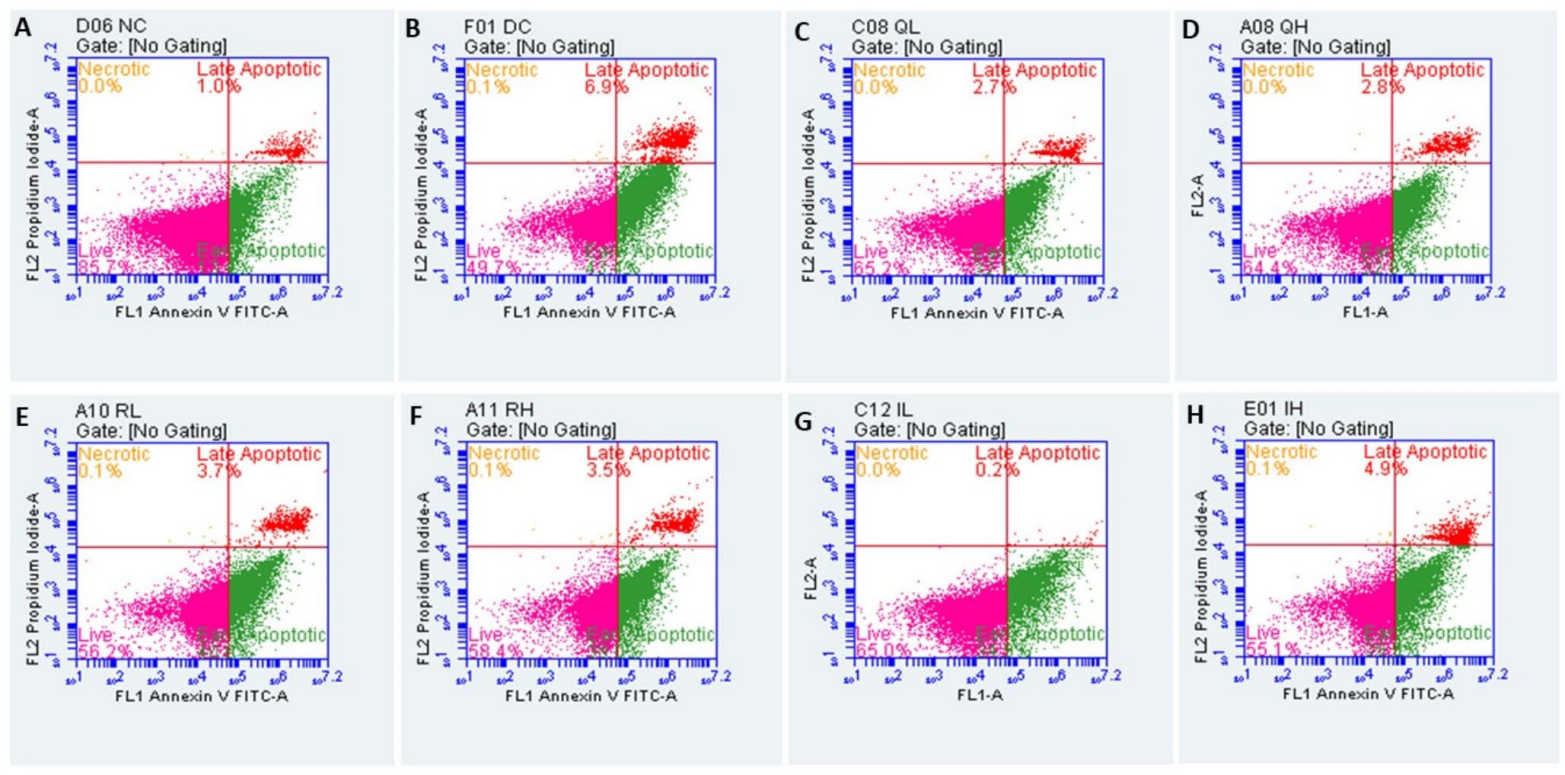
Effect of QRT, RUT and IQRT concentrations on apoptotic phases upon MF-induced toxicity after FITC annexin v/propidium iodide apoptosis assay in differentiated SH-SY5Y cells. **A**: Normal Control; **B**: MF Control; **C**: Quercetin (0.12 µM); **D**: Quercetin (0.6 µM); **E**: Rutin (16 µM); **F**: Rutin (80 µM); **G**: Isoquercetin (16 µM); **H**: Isoquercetin (80 µM). Images are representations of three independent experiments

**Fig. 9:**
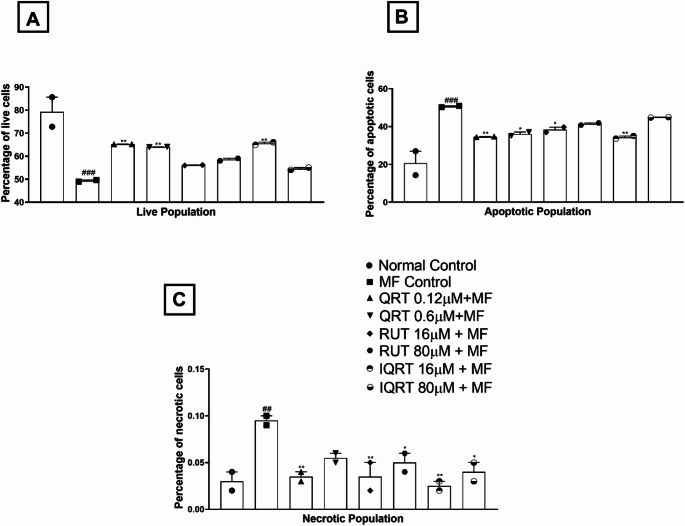
Effect of QRT, RUT and IQRT concentrations on apoptotic phases **A**: Live; **B**: Apoptotic and **C**: Necrotic population upon MF-induced toxicity after FITC annexin v/propidium iodide apoptosis assay in differentiated SH-SY5Y cells. Data represents mean ± SEM of two independent experiments (*n* = 2) analysed by One-way ANOVA followed by Dunnet’s post-hoc test. ###*p* < 0.001,##*p* < 0.01 compared to normal control group, **p < 0.01, *p < 0.05 compared to the MF control group. (QRT: Quercetin; RUT: Rutin; IQRT: Isoquercetin)

#### Effect of quercetin and its analogues on mRNA levels of NAMPT and SIRT1 genes in differentiated SH-SY5Y cells

**Fig. 10:**
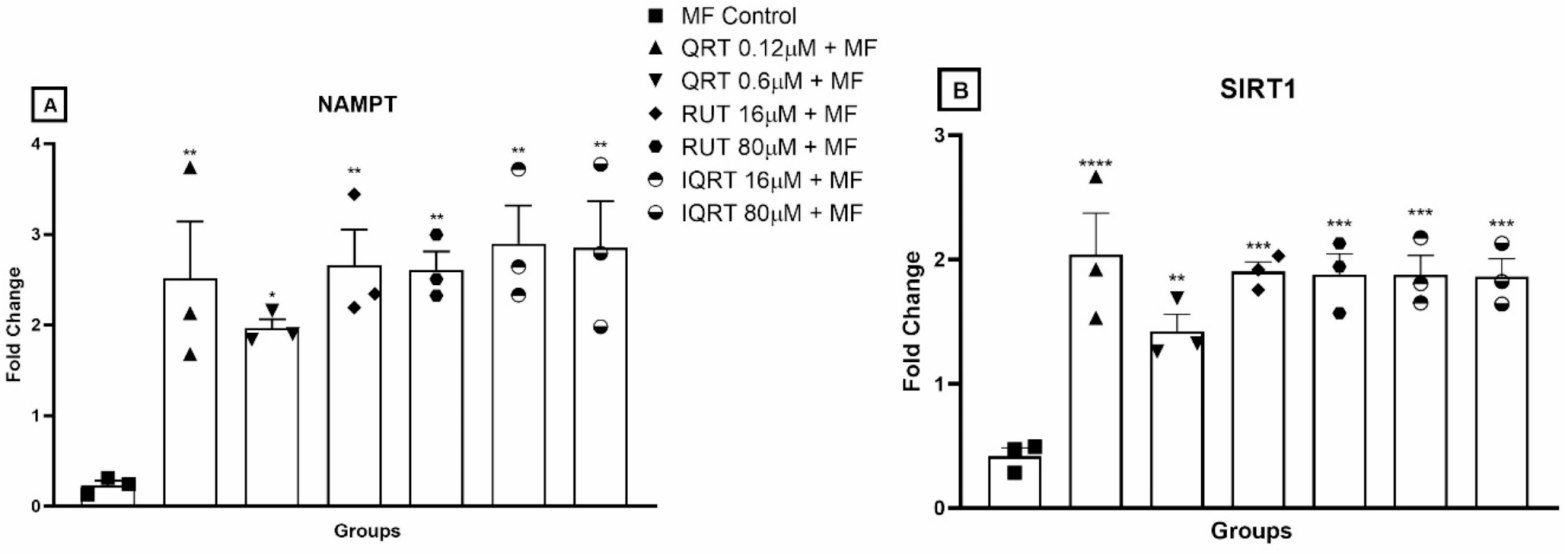
Effect of different QRT, RUT, and IQRT concentrations on **A**: NAMPT; **B**: SIRT1 mRNA expression upon MF-induced toxicity in differentiated SH-SY5Y cells. Data represents mean ± SEM of three independent experiments (*n* = 3) analysed by One-way ANOVA followed by Dunnet’s post-hoc test. *p < 0.05,**p < 0.01,***p < 0.001,****p < 0.0001 compared to MF control group. (QRT: Quercetin; RUT: Rutin; IQRT: Isoquercetin)

RT-PCR analysis was performed to determine the changes in mRNA levels of target genes, NAMPT and SIRT1, after pretreatment with the test compounds in differentiated SH-SY5Y cells. One-way ANOVA revealed a statistically significant difference in the treatment groups’ target gene expressions of NAMPT (_F6,14_=5.944, *p* = 0.0029) and SIRT1 (F6,14 = 10.69, *p* = 0.0002). For NAMPT, quercetin at 0.12 µM (2.518 ± 0.6244 vs. 0.2296 ± 0.05409, *p* < 0.01), 0.6 µM (1.966 ± 0.09808 vs. 0.2296 ± 0.05409, *p* < 0.05), rutin at 16 µM (2.660 ± 0.3938 vs. 0.2296 ± 0.05409, *p* < 0.01), 80 µM (2.610 ± 0.2009 vs. 0.2296 ± 0.05409, *p* < 0.01) and isoquercetin at 16 µM (2.898 ± 0.4200 vs. 0.2296 ± 0.05409, *p* < 0.001), 80 µM (2.849 ± 0.5179 vs. 0.2296 ± 0.05409, *p* < 0.001) increased the expression of NAMPT in the neuronal cells as compared to the MF-treated group. For SIRT1, quercetin at 0.12 µM (2.040 ± 0.3329 vs. 0.4170 ± 0.06672, *p* < 0.0001), 0.6 µM (1.423 ± 0.1333 vs. 0.4170 ± 0.06672, *p* < 0.01), rutin at 16 µM (1.900 ± 0.07892 vs. 0.4170 ± 0.06672, *p* < 0.001), 80 µM (1.880 ± 0.1652 vs. 0.4170 ± 0.06672, *p* < 0.001) and isoquercetin at 16 µM (1.876 ± 0.1554 vs. 0.4170 ± 0.06672, *p* < 0.001), 80 µM (1.864 ± 0.1425 vs. 0.4170 ± 0.06672, *p* < 0.001) increased the expression of SIRT1 in the neuronal cells as compared to the MF-treated group. All doses of test drug treatments showed a significant increase in the expression of target genes compared to the MF control group, which implies that the activation of NAMPT and SIRT1 may play an important role in counteracting the MF-induced neurotoxicity in the neuronal cells (Fig. 10).

## Discussion

The increasing concern about chemobrain among cancer patients/survivors made it to be recognised as a clinical syndrome because of its negative impact on their quality of life. Also, the non-availability of drugs demands a comprehensive understanding of the underlying mechanisms to identify suitable drug targets that can prevent and cure the disease through nutrition.

Our in silico study attempts to characterise proteins like NAMPT and SIRT1 that may have a possible role in modulating chemobrain. Here, the role of quercetin and its few natural derivatives, already proven preclinically for their neuroprotective potential (Butterweck et al. 2004; Mandel et al. 2005; Balez et al. 2016; Inoue et al. 2019; Jamali-Raeufy et al. 2019; Çelik et al. 2020; Tan et al. 2020; Fan et al. 2021; Yang et al. 2021; Hamdi et al. 2021; Hong et al. 2022), was evaluated in activating NAMPT and SIRT1 proteins.

Sirtuins, essential anti-aging factors, are conserved across all living organisms with nearly identical structures and functions (Vassilopoulos et al. 2011). SIRT1 is a well-studied mammalian sirtuin that plays a crucial role in cellular processes. The N-terminal domain (NTD), which has three helices, the HDAC domain, and a β-hairpin from the C-terminal regulatory segment (CTR), also known as the region required for SIRT1 activity, make up the three main portions of full-length SIRT1. The NTD is alternatively referred to as sirtuin-activating compounds (STACs) binding domains or SBD. Dai et al. explained how different STACs activate SIRT1. They proposed that the binding of STACs and the initiation of deacetylation are the functions of the NTD. Furthermore, their research postulated that GLU230’s negative charge stabilises human SIRT1’s activated conformation, which interacts with a positively charged residue (Dai et al. 2015). Other studies have also reported that the allosteric site activates SIRT1 (Hubbard et al. 2013; Bakhtiari et al. 2018; Manjula et al. 2021).

Based on evidence from the literature, we found that GLU230 is crucial for activating the SIRT1 protein, as shown by most selected compounds. So, considering the dock score, other amino acid interactions, and stability, we have found that derivatives like Rutin, Isoquercetin, Miquelianin, and Hyperoside can be selected for performing molecular dynamic studies along with quercetin. So, in the 20ns time frame, except for quercetin and hyperoside, the rest of the ligands showed a very stable complex, which can be seen in their RMSD values throughout the MD simulation. In this present study, in silico docking and simulation studies of SIRT1 with quercetin and its derivatives showed the potential ability of these compounds as direct SIRT1-activating compounds.

NAD + is a crucial chemical that is involved in several biological activities. It functions as a signaling molecule and cofactor in many redox processes. Alzheimer’s, Parkinson’s, and amyotrophic lateral sclerosis are among the neurodegenerative illnesses associated with low NAD + levels in the aging brain. Nearly every facet of neuronal aging is influenced by NAD+(Lautrup et al. 2019).

Many studies have been carried out on the anticancer actions of NAMPT with the accessibility of cocrystal structures of NAMPT with different NAMPT inhibitors (Zak et al. 2015; Wilsbacher et al. 2017; Tang et al. 2023) On the other hand, even though the evidence exists stating the neuroprotective actions of NAMPT along with the discovery of NAMPT activators like P7C3 and SBI-797,812, cocrystal structures of NAMPT with the activator were not available for the initial validation of possible activators of NAMPT through molecular simulations studies (Wang et al. 2014; Gardell et al. 2019). Therefore, given that NAMPT plays a crucial part in neuroprotection via the NAD + salvage route, it is imperative to find new small compounds that specifically target NAMPT to increase NAD + production. Chinese researchers recently discovered the cocrystal structure of a small molecule called activator NAT coupled to NAMPT, triggering a confirmational change via the condensation of NAM and PRPP to create Nicotinamide mononucleotide (NMN). The experiments conducted both in vitro and in vivo revealed that this small molecule increases the enzymatic activity of NAMPT in a robust and targeted manner. The researchers postulated that NAT has an allosteric activating effect on NAMPT based on the cocrystal structures of NAT and NAMPT. By binding and changing the configuration of amino acid residues close to the active site, the activity of the NAMPT enzyme is increased (Yao et al. 2022). Here, we have docked the quercetin and its different natural derivatives in the place of NAT and compared its interactions with the ligands of interest. So, for NAMPT protein, based on the docking score, amino acid interactions, particularly LYN189, ASP219, SER275, VAL242, and stability, derivatives like Rutin, Isoquercetin, Miquelianin and Hyperoside were selected for performing molecular dynamics along with quercetin. So, in the selected trajectory of 20ns, all ligands and quercetin formed stable complexes with the protein shown by the least change in displacement of atoms indicated by the acceptable range of their RMSD values. Apart from this, the non-availability of co-crystallised structures of the NAMPT with the activator molecule makes it difficult to arrive at a broader conclusion, especially on the interactions, energy, and structural deviations from the protein.

After evaluating several predictions regarding stability and interactions with target proteins, the crucial factor is determining the potential of the compounds. Based on the collective data from all the predictors, rutin and isoquercetin were chosen for further screening through in vitro experimentation.

Hippocampal neurons are central memory formation (Kolibius et al. 2023). Chemotherapeutic agents are more toxic to hippocampal cells than cancer cells (Dietrich et al. 2006; Kovalchuk and Kolb 2017). Studies have documented that methotrexate and 5-fluorouracil harm memory by compromising neuronal proliferation in the hippocampus (Winocur et al. 2006; Foley et al. 2008). Hence, differentiated SH-SY5Y cells were utilised for the study to evaluate the neurotoxic potential of MF and overcome the challenges associated with culturing the neuronal cells. The primary step for the in vitro study was identifying IC_50_ values of methotrexate, 5-fluorouracil, quercetin, rutin, and isoquercetin. Treatment with methotrexate and 5-fluorouracil combined showed more cytotoxicity than when treated individually. Rutin and isoquercetin showed IC_50_ values greater than 2000 µM, whereas quercetin showed around 60 µM. This aligns with the previous evidence where in vitro studies show that protective effects were obtained with quercetin at lower concentrations (25 and 50 µM). In contrast, toxic effects are observed with concentrations exceeding 100 µM. Cytoprotective effects of quercetin in neurodegenerative models usually occur at lower concentrations than its DNA-damaging and proapoptotic actions (Ossola et al. 2008; Dajas 2012).

The neuron-like phenotype in SH-SY5Y was accomplished by treatment with retinoic acid (10 µM). Individual doses of quercetin and its analogues produced more than 70% viability and were selected for assessing their neuroprotective potential in combination with the toxicants. In the presence of retinoic acid, the cell viability at all doses of the drugs was increased by approximately 20% compared to that in undifferentiated cells. The cytotoxicity produced by MF was reduced from 50 to 44% in differentiated cells. So, the increase of antiapoptotic Bcl-2 protein and survival signaling through activating the phosphatidylinositol 3-kinase/Akt signaling pathway is responsible for the difference in cell viability between differentiated and undifferentiated cells. Prior research suggests that retinoic acid-induced differentiated cells are more resilient than undifferentiated cells to toxin-induced cell death caused by toxicants such as glutamate, 6-hydroxydopamine, 1-methyl-4-phenyl-1,2,3,6-tetrahydropyridine, or its metabolite, 1-methyl-4-phenylpyridinium ion (Cheung et al. 2009; Nampoothiri et al. 2014). In the present study, we found that undifferentiated SH-SY5Y cells were susceptible to MF, but RA-induced differentiation conferred higher tolerance to SH-SY5Y cells and reduced MF toxicity.

In vitro investigation showed that MF has neurotoxic potential in SH-SY5Y cells, resulting in reduced neurite length and neuronal damage compared to normal control cells. Also, the treatment of quercetin and its analogues before the MF challenge has protected the cells from neuronal damage, evidenced by the increased neurite length and viability. Quercetin showed maximum neurite length at 0.12 µM and 0.6 µM, Rutin and Isoquercetin at 16 µM and 80 µM, after which neurite length was found to be almost similar and did not increase significantly.

Morphological alterations during apoptosis in SH-SY5Y cells as a result of MF and test drug treatments were evaluated by AO/EB staining. Previous studies have shown that quercetin is an effective inhibitor of the caspase cascade, responsible for DNA fragmentation, leading to apoptosis and cell death. Flavonoids activate PI3/Akt and ERK signalling pathways, which stimulate antiapoptotic gene expression, such as Bcl-2 and X-linked inhibitors of apoptosis protein, resulting in neuroprotective effects (Suematsu et al. 2011; Lei et al. 2015; Zhai et al. 2016). In AO/EB staining, quercetin and analogues significantly reversed the MF-induced apoptotic damage in the cells, demonstrating the antiapoptotic potential of the flavonoids at the selected doses in the neuronal cells.

In addition, flow cytometric analysis using Annexin V-FITC/PI staining revealed how different doses of the phytochemicals respond in various stages of apoptosis after the MF challenge. The tested concentration of MF promoted early and late apoptosis with significant cell death in the necrotic phase compared to the untreated cells. The lower percentage of the necrotic population than the apoptotic population may be because, at lower doses, anticancer drugs can only induce apoptosis. Still, the same drugs may cause necrosis at higher doses (Elmore 2007). In addition, the anticancer strategy is generally meant to induce apoptosis and not necrosis due to the deleterious release of toxic components (Lekshmi et al. 2017). However, the impairment of phagocytosis marks the transition from early to late apoptotic phase, resulting in increased permeability (Poon et al. 2009).The cells exposed to the later phase of apoptosis release proinflammatory cytokines like IL-6 and TNF-α which may be responsible for the neuroinflammatory properties of methotrexate and 5-fluorouracil that can be attributed to their cognitive impairment effects shown in different preclinical and clinical studies (John et al. 2021, 2022). The chemotherapeutic properties of methotrexate and 5-fluorouracil are attributed to mitochondrial ROS generation and the caspase cascade activation (Sarder et al. 2015; Mavrikou et al. 2019). The proapoptotic nature of methotrexate and 5-fluorouracil induced increased cells in the early apoptotic phase, which was reversed by the drug treatments. The quercetin and analogues were found to protect the cells by preventing their progression to the later phase of apoptosis. However, the actual mechanism of methotrexate and 5-fluorouracil in apoptosis induction has yet to be elucidated. Intracellular oxidation or oxidative stress is one of the factors that can trigger apoptosis (Herman et al. 2005). Interestingly, antioxidants can also protect the cell from apoptosis, even when caused by stimuli that do not directly affect the cell through oxidation (Kane et al. 1993). It has been reported that TNF-α, required for host defence, exerts its cytotoxic effect by generating intracellular ROS that induce apoptosis (Hirose et al. 1993; Schulze-Osthoff et al. 1994). Therefore, the cytotoxic anti-proliferative effect of methotrexate and 5-fluorouracil may be accompanied by apoptotic manifestations or enhanced ROS generation, and the antiapoptotic nature of quercetin, rutin, and isoquercetin may be attributed to their antioxidant defence mechanisms. To substantiate this, further studies on ROS are warranted.

Cells pretreated with quercetin and its analogues were evaluated for changes in mRNA levels of NAMPT and SIRT1 proteins. It was found that treatment of MF in cells showed 4.355 times and 2.398 times downregulation in mRNA levels of NAMPT and SIRT1 proteins, respectively. Quercetin at 0.12 µM and 0.6 µM showed upregulation in mRNA levels of NAMPT by 10.966 times and 8.633 times, while for SIRT1, it was 4.892 times and 3.4127 times, respectively. Rutin at 16 µM and 80 µM showed upregulation in mRNA levels of NAMPT by 11.585 times and 11.367 times, while for SIRT1, it was 4.556 times and 4.508 times, respectively. Isoquercetin at 16 µM and 80 µM showed upregulation in mRNA levels of NAMPT by 12.621 times and 12.408 times, while for SIRT1, it was 4.498 times and 4.470 times, respectively. Yoo et al. have recently reported that supplementation of nicotinamide mononucleotide prevented cisplatin-induced cognitive impairment in female adult mice. The researchers demonstrated that cisplatin can reduce the production of NAD + and NAMPT in human cortical neurons (Yoo et al. 2021). NAD + and NAMPT are involved in the activation of SIRT1. NAMPT regulates SIRT1 activity through the control of NAD + concentrations. Moreover, NAD + enhances the energy supply within cells and prevents apoptosis (Van Der Veer et al. 2007; Zhang et al. 2009). SIRT1 is crucial in decreasing oxidative stress, stimulating antioxidant defenses, and preventing apoptosis (Kiss et al. 2019). Similarly, NAMPT inhibitors are found to induce apoptosis in cancer cells (Amjad et al. 2021), and so are chemotherapeutic agents. Altogether, the induction of apoptotic processes by the MF treatment could have significantly inhibited NAMPT and SIRT1 expression in the neuronal cells. In contrast, the antiapoptotic potential of quercetin, rutin, and isoquercetin possibly enhanced the NAMPT and SIRT1 expression and, thereby reversing the neurotoxic insult induced by the MF treatment. Hence, our study identifies the possible role of MF in disrupting the NAMPT-mediated NAD+/SIRT1 axis in SH-SY5Y cells. Therefore, prior treatment of quercetin, rutin, and isoquercetin demonstrated their protective effect against MF-induced neurotoxicity and the possibility of acting as either NAMPT/SIRT1 activators to maintain NAD + levels.

## Conclusion

Phytochemicals have received considerable focus regarding neuroprotection, especially in cases like chemobrain, where cognitive decline results from oxidative stress, inflammation, and metabolic irregularities. Our research emphasises the importance of NAMPT and SIRT1—crucial factors in cellular metabolism and neuroprotection—in the possible therapeutic effects of phytochemicals. NAMPT plays a vital role in the production of NAD⁺, which enhances SIRT1 function. SIRT1, in turn, influences neuroinflammation, mitochondrial performance, and synaptic plasticity, all of which are compromised in chemobrain. Our results indicate the potential for phytochemical-based therapies in managing chemobrain. Additional preclinical and clinical research is needed to confirm these mechanisms in human subjects. Our in vitro study may serve as a primary step in understanding NAMPT and SIRT1 in the chemo brain and identifying possible compounds for activating these proteins, which might help mitigate long-term side effects caused by chemotherapeutic agents.

## Limitations

- The number of naturally occurring analogues of quercetin selected for screening through in silico analysis was limited in this study. Therefore, the inclusion of a large number of compounds related to quercetin is recommended to derive more compounds through drug simulation studies.
- To fully assess the bioavailability of quercetin, it is necessary to conduct chronic in vivo studies. This may be because acute quercetin administration cannot reach the effective threshold of pharmacological plasma concentration. Therefore, the active concentration of quercetin applied in vitro cannot be translated directly into in vivo situations.
- NAD + activity assessment is essential to establish the involvement of NAMPT-mediated NAD+/SIRT1 in CICI.
- It is also crucial to confirm the levels of NAMPT and SIRT1 in tumor cells to confirm the role of these proteins in the presence and absence of chemotherapy.
- Future studies should investigate the long-term effects of sustained NAMPT/SIRT1 activation, including potential metabolic imbalances, neuronal stress responses, and compensatory mechanisms.
- It is necessary to investigate the potential of quercetin to prevent or reduce the onset of chemobrain if administered prophylactically by designing RCTs and longitudinal studies to study the long-term effects of quercetin on cognitive function and its potential role in the recovery from chemobrain.

## Electronic supplementary material

Below is the link to the electronic supplementary material.

Supplementary Material 1

## Data Availability

Datasets are available from the corresponding author on reasonable request.

## Abbreviations

AO/EB: Acridine Orange/Ethidium Bromide
ARG: Arginine
ASN: Asparagine
ASP: Aspartic acid
Bcl-2: B-cell Lymphoma 2
CICI: Chemotherapy-induced cognitive impairment
CTR: C-Terminal Domain
ERK: Extracellular signal-regulated kinase
FITC: Fluorescein Isothiocyanate
GLN: Glutamine
GLU: Glutamic acid
HDAC: Histone deacetylases
HIS: Histidine
IC50: Half-maximal inhibitory concentration
ID: Identification
IL-6: Interleukin-6
ILE: Isoleucine
IQRT: Isoquercetin
LEU: Leucine
MD: Molecular Dynamics
MF: Methotrexate and 5-Fluorouracil
MM-GBSA: Molecular mechanics with generalised Born and surface area solvation
NAD+: Nicotinamide adenine dinucleotide
NAM: Nicotinamide
NAMPT: Nicotinamide phosphoribosyl transferase
NCCS: National Centre for Cell Science
NMN: Nicotinamide Mononucleotide
NTD: N-Terminal Domain
OPLS3e: Optimized Potentials for Liquid Simulations 3 Extended
PBS: Phosphate Buffered Saline
PDB: Protein Data Bank
PI: Propidium iodide
PI3/Akt: Phosphatidylinositol 3-Kinase/Akt
PRO: Proline
PRPP: Phosphoribosyl pyrophosphate
RA: Retinoic acid
RCSB: Research Collaboratory for Structural Bioinformatics
RMSD: Root Mean Square Deviation
ROS: Reactive oxygen species
RT-PCR: Real time-Polymerase Chain Reaction
RUT: Rutin
SBD: STAC Binding Domain
SER: Serine
SIRT1: Sirtuin-1
SRB: Sulforhodamine-B
STACs: Sirtuin-Activating Compounds
THR: Threonine
TNF-α: Tumor Necrosis Factor-alpha
TYR: Tyrosine
QRT: Quercetin
VAL: Valine

## References

[CR1] Ahles TA, Root JC, Ryan EL (2012) Cancer- and cancer treatment-associated cognitive change: an update on the state of the science. J Clin Oncol 30:3675–3686. 10.1200/JCO.2012.43.0116 23008308 10.1200/JCO.2012.43.0116 PMC3675678

[CR2] Amjad S, Nisar S, Bhat AA et al (2021) Role of NAD + in regulating cellular and metabolic signaling pathways. Mol Metab 49:101195. 10.1016/J.MOLMET.2021.101195 33609766 10.1016/j.molmet.2021.101195 PMC7973386

[CR3] Bakhtiari N, Mirzaie S, Hemmati R et al (2018) Mounting evidence validates ursolic acid directly activates SIRT1: A powerful STAC which mimic endogenous activator of SIRT1. Arch Biochem Biophys 650:39–48. 10.1016/j.abb.2018.05.012 29758202 10.1016/j.abb.2018.05.012

[CR4] Bakoyiannis I, Daskalopoulou A, Pergialiotis V, Perrea D (2019) Phytochemicals and cognitive health: are flavonoids doing the trick? Biomed Pharmacother 109:1488–1497. 10.1016/J.BIOPHA.2018.10.086 30551400 10.1016/j.biopha.2018.10.086

[CR5] Balez R, Steiner N, Engel M et al (2016) Neuroprotective effects of apigenin against inflammation, neuronal excitability and apoptosis in an induced pluripotent stem cell model of Alzheimer’s disease. Sci Rep 6. 10.1038/SREP31450 10.1038/srep31450 PMC4981845 27514990

[CR6] Bi TQ, Che XM, Liao XH et al (2011) Overexpression of Nampt in gastric cancer and chemopotentiating effects of the Nampt inhibitor FK866 in combination with fluorouracil. Oncol Rep 26:1251–1257. 10.3892/or.2011.1378 21743967 10.3892/or.2011.1378

[CR7] Butterweck V, Hegger M, Winterhoff H (2004) Flavonoids of St. John’s wort reduce HPA axis function in the rat. Planta Med 70:1008–1011. 10.1055/S-2004-832631 15490333 10.1055/s-2004-832631

[CR8] Çelik H, Fatih ·, Kandemir M et al (2020) Neuroprotective effect of Rutin against colistin-induced oxidative stress, inflammation and apoptosis in rat brain associated with the CREB/BDNF expressions. Mol Biol Rep 47:2023–2034. 10.1007/s11033-020-05302-z 32030599 10.1007/s11033-020-05302-z

[CR9] Chang L, Weiner LS, Hartman SJ et al (2018) Breast cancer treatment and its effects on aging. J Geriatr Oncol 10:346–355. 10.1016/j.jgo.2018.07.010 30078714 10.1016/j.jgo.2018.07.010 PMC7062379

[CR10] Cheung YT, Lau WKW, Yu MS et al (2009) Effects of all-trans-retinoic acid on human SH-SY5Y neuroblastoma as in vitro model in neurotoxicity research. Neurotoxicology 30:127–135. 10.1016/J.NEURO.2008.11.001 19056420 10.1016/j.neuro.2008.11.001

[CR11] Costa LG, Garrick JM, Roquè PJ, Pellacani C (2016) Mechanisms of neuroprotection by Quercetin: counteracting oxidative stress and more. Oxid Med Cell Longev. 10.1155/2016/2986796 26904161 10.1155/2016/2986796 PMC4745323

[CR12] Cupit-Link MC, Kirkland JL, Ness KK et al (2017) Biology of premature ageing in survivors of cancer. ESMO Open 2:e000250. 10.1136/ESMOOPEN-2017-000250 29326844 10.1136/esmoopen-2017-000250 PMC5757468

[CR13] Dai H, Case AW, Riera TV et al (2015) Crystallographic structure of a small molecule SIRT1 activator-enzyme complex. Nat Commun 6:1–10. 10.1038/ncomms8645 10.1038/ncomms8645 PMC4506539 26134520

[CR14] Dajas F (2012) Life or death: neuroprotective and anticancer effects of Quercetin. J Ethnopharmacol 143:383–396. 10.1016/J.JEP.2012.07.005 22820241 10.1016/j.jep.2012.07.005

[CR15] Dietrich J, Han R, Yang Y et al (2006) CNS progenitor cells and oligodendrocytes are targets of chemotherapeutic agents in vitro and in vivo. J Biol 5. 10.1186/JBIOL50 10.1186/jbiol50 PMC2000477 17125495

[CR16] Elmore S (2007) Apoptosis: A review of programmed cell death. Toxicol Pathol 35:495–516 17562483 10.1080/01926230701320337 PMC2117903

[CR17] Fan H, Li Y, Sun M et al (2021) Hyperoside reduces Rotenone-induced neuronal injury by suppressing autophagy. Neurochem Res 46:3149–3158. 10.1007/s11064-021-03404-z 10.1007/s11064-021-03404-z 34415495

[CR18] Foley JJ, Raffa RB, Walker EA (2008) Effects of chemotherapeutic agents 5-fluorouracil and methotrexate alone and combined in a mouse model of learning and memory. Psychopharmacology 199:527. 10.1007/S00213-008-1175-Y 18463849 10.1007/s00213-008-1175-y PMC3263345

[CR19] Gardell SJ, Hopf M, Khan A et al (2019) Boosting NAD + with a small molecule that activates NAMPT. Nat Commun 10. 10.1038/S41467-019-11078-Z 10.1038/s41467-019-11078-z PMC6642140 31324777

[CR20] Genheden S, Ryde U (2015) Expert opinion on drug discovery the MM/PBSA and MM/GBSA methods to estimate ligand-binding affinities. Expert Opin Drug Discov 10:449–461. 10.1517/17460441.2015.1032936 25835573 10.1517/17460441.2015.1032936 PMC4487606

[CR21] Hamdi H, Abid-Essefi S, Eyer J (2021) Neuroprotective effects of myricetin on Epoxiconazole-induced toxicity in F98 cells. Free Radic Biol Med 164:154–163. 10.1016/J.FREERADBIOMED.2020.12.451 33429020 10.1016/j.freeradbiomed.2020.12.451

[CR22] Herman S, Zurgil N, Deutsch M (2005) Low dose methotrexate induces apoptosis with reactive oxygen species involvement in T lymphocytic cell lines to a greater extent than in monocytic lines. Inflamm Res 54:273–280. 10.1007/S00011-005-1355-8 16134056 10.1007/s00011-005-1355-8

[CR23] Hill A, Sadda J, Labarge MA, Hurria A (2019) How cancer therapeutics cause accelerated aging: insights from the hallmarks of aging. J Geriatr Oncol 11:191–193. 10.1016/j.jgo.2019.03.007 30905710 10.1016/j.jgo.2019.03.007

[CR24] Hirose K, Longo DL, Oppenheim JJ, Matsushima K (1993) Overexpression of mitochondrial manganese superoxide dismutase promotes the survival of tumor cells exposed to interleukin-1, tumor necrosis factor, selected anticancer drugs, and ionising radiation. FASEB J 7:361–368. 10.1096/FASEBJ.7.2.8440412 8440412 10.1096/fasebj.7.2.8440412

[CR25] Ho CL, Kao NJ, Lin CI et al (2022) Quercetin increases mitochondrial biogenesis and reduces free radicals in neuronal SH-SY5Y cells. 10.3390/nu14163310. Nutrients 14:10.3390/nu14163310 PMC9414536 36014814

[CR26] Hong DG, Lee S, Kim J et al (2022) Anti-Inflammatory and Neuroprotective Effects of Morin in an MPTP-Induced Parkinson’s Disease Model. Int J Mol Sci 23. 10.3390/IJMS231810578 10.3390/ijms231810578 PMC9501291 36142491

[CR27] Huang W, Chen C, Sze C, Teng C (2013) Visfatin induces stromal Cell-Derived Factor-1 expression by b1 integrin signaling in colorectal Cancer cells. J Cell Physiol 228:1017–1024. 10.1002/jcp.24248 23042611 10.1002/jcp.24248

[CR28] Hubbard BP, Gomes AP, Dai H et al (2013) Evidence for a common mechanism of SIRT1 regulation by allosteric activators. Science (1979) 339:1216–1219. 10.1126/science.1231097 10.1126/science.1231097 PMC3799917 23471411

[CR29] Inoue T, Saito S, Tanaka M et al (2019) Pleiotropic neuroprotective effects of taxifolin in cerebral amyloid angiopathy. Proc Natl Acad Sci USA 116:10031–10038. 10.1073/PNAS.1901659116 31036637 10.1073/pnas.1901659116 PMC6525485

[CR30] Jamali-Raeufy N, Baluchnejadmojarad T, Roghani M et al (2019) Isorhamnetin exerts neuroprotective effects in STZ-induced diabetic rats via Attenuation of oxidative stress, inflammation and apoptosis. J Chem Neuroanat 102. 10.1016/J.JCHEMNEU.2019.101709 10.1016/j.jchemneu.2019.101709 31698018

[CR31] Janelsins MC, Heckler CE, Peppone LJ et al (2017) Cognitive complaints in survivors of breast cancer after chemotherapy compared with Age-Matched controls: an analysis from a nationwide, multicenter, prospective longitudinal study. J Clin Oncol 35:506–514. 10.1200/JCO.2016.68.5826 28029304 10.1200/JCO.2016.68.5826 PMC5455314

[CR32] Jin T, Zhang Y, Botchway BOA et al (2023) Quercetin activates the Sestrin2/AMPK/SIRT1 axis to improve amyotrophic lateral sclerosis. Biomed Pharmacother 161:114515. 10.1016/J.BIOPHA.2023.114515 36913894 10.1016/j.biopha.2023.114515

[CR33] John J, Kinra M, Mudgal J et al (2021) Animal models of chemotherapy-induced cognitive decline in preclinical drug development. Psychopharmacology 238:3025–3053. 10.1007/s00213-021-05977-7 34643772 10.1007/s00213-021-05977-7 PMC8605973

[CR34] John J, Kinra M, Ranadive N et al (2022) Neuroprotective effect of Mulmina Mango against chemotherapy-induced cognitive decline in mouse model of mammary carcinoma. Sci Rep 12:1–12. 10.1038/s41598-022-06862-9 35197512 10.1038/s41598-022-06862-9 PMC8866531

[CR35] Kane DJ, Sarafian TA, Anton R et al (1993) Bcl-2 inhibition of neural death: Decreased generation of reactive oxygen species. Science (1979) 262:1274–1277. 10.1126/SCIENCE.8235659 10.1126/science.8235659 8235659

[CR36] Keni R, Nayak PG, Kumar N et al (2023) Sesamol combats diabetogenic effects of Atorvastatin through GLUT-4 expression and improved pancreatic viability. 3 Biotech 13:1–11. 10.1007/S13205-023-03784-9/METRICS 10.1007/s13205-023-03784-9 PMC10597939 37885753

[CR37] Kennedy BE, Sharif T, Martell E et al (2016) NAD + salvage pathway in cancer metabolism and therapy. Pharmacol Res 114:274–283 27816507 10.1016/j.phrs.2016.10.027

[CR38] Kincaid JWR, Berger NA (2020) NAD metabolism in aging and cancer. Exp Biol Med 245:1594–1614 10.1177/1535370220929287 PMC7787546 32500741

[CR39] Kiss T, Giles CB, Tarantini S et al (2019) Nicotinamide mononucleotide (NMN) supplementation promotes anti-aging MiRNA expression profile in the aorta of aged mice, predicting epigenetic rejuvenation and anti-atherogenic effects. Geroscience 41:419–439. 10.1007/S11357-019-00095-X 31463647 10.1007/s11357-019-00095-x PMC6815288

[CR40] Kolibius LD, Roux F, Parish G et al (2023) Hippocampal neurons code individual episodic memories in humans. Nat Hum Behav 2023 7:11. 10.1038/s41562-023-01706-6 10.1038/s41562-023-01706-6 PMC10663153 37798368

[CR41] Kovalchuk A, Kolb B (2017) Chemo brain: from discerning mechanisms to lifting the brain fog—An aging connection. Cell Cycle 16:1345–1349. 10.1080/15384101.2017.1334022 28657421 10.1080/15384101.2017.1334022 PMC5539816

[CR42] Lautrup S, Sinclair DA, Mattson MP, Fang EF (2019) NAD + in brain aging and neurodegenerative disorders. Cell Metab 30:630–655. 10.1016/J.CMET.2019.09.001 31577933 10.1016/j.cmet.2019.09.001 PMC6787556

[CR43] Lei X, Chao H, Zhang Z et al (2015) Neuroprotective effects of Quercetin in a mouse model of brain ischemic/reperfusion injury via antiapoptotic mechanisms based on the Akt pathway. Mol Med Rep 12:3688–3696. 10.3892/mmr.2015.3857 26016839 10.3892/mmr.2015.3857

[CR44] Lekshmi A, Varadarajan SN, Lupitha SS et al (2017) A quantitative real-time approach for discriminating apoptosis and necrosis. Cell Death Discov 3. 10.1038/cddiscovery.2016.101 10.1038/cddiscovery.2016.101 PMC5253725 28179996

[CR45] Livak KJ, Schmittgen TD (2001) Analysis of relative gene expression data using real-time quantitative PCR and the 2(-Delta Delta C(T)) method. Methods 25:402–408. 10.1006/METH.2001.1262 11846609 10.1006/meth.2001.1262

[CR46] Maldi E, Travelli C, Caldarelli A et al (2013) Nicotinamide phosphoribosyltransferase (NAMPT) is over-expressed in melanoma lesions. Pigment Cell Melanoma Res 26:144–146 23051650 10.1111/pcmr.12037

[CR47] Mandel SA, Avramovich-Tirosh Y, Reznichenko L et al (2005) Multifunctional activities of green tea catechins in neuroprotection. Modulation of cell survival genes, iron-dependent oxidative stress and PKC signaling pathway. Neurosignals 14:46–60. 10.1159/000085385 15956814 10.1159/000085385

[CR48] Manjula R, Anuja K, Alcain FJ (2021) SIRT1 and SIRT2 activity control in neurodegenerative diseases. Front Pharmacol 11:585821. 10.3389/fphar.2020.585821 10.3389/fphar.2020.585821 PMC7883599 33597872

[CR49] Mavrikou S, Tsekouras V, Karageorgou MA et al (2019) Detection of superoxide alterations induced by 5-Fluorouracil on HeLa cells with a Cell-Based biosensor. Biosens (Basel) 9. 10.3390/BIOS9040126 10.3390/bios9040126 PMC6956086 31623083

[CR50] n Der Veer E, Ho C, O’Neil C et al (2007) Extension of human cell lifespan by nicotinamide phosphoribosyltransferase. J Biol Chem 282:10841–10845. 10.1074/JBC.C700018200 17307730 10.1074/jbc.C700018200

[CR51] Nampoothiri M, Reddy ND, John J et al (2014) Insulin blocks Glutamate-Induced neurotoxicity in differentiated SH-SY5Y neuronal cells. 10.1155/2014/674164. Behavioural neurology 2014:10.1155/2014/674164 PMC4082871 25018588

[CR52] Nguyen LD, Ehrlich BE (2020) Cellular mechanisms and treatments for chemobrain: insight from aging and neurodegenerative diseases. EMBO Mol Med 12:e12075. 10.15252/EMMM.202012075 32346964 10.15252/emmm.202012075 PMC7278555

[CR53] Ossola B, Kääriäinen TM, Raasmaja A, Männistö PT (2008) Time-dependent protective and harmful effects of Quercetin on 6-OHDA-induced toxicity in neuronal SH-SY5Y cells. Toxicology 250:1–8. 10.1016/J.TOX.2008.04.001 18756631 10.1016/j.tox.2008.04.001

[CR54] Pemberton K, Mersman B, Xu F (2018) Using ImageJ to assess neurite outgrowth in mammalian cell cultures: research data quantification exercises in undergraduate neuroscience lab. J Undergrad Neurosci Educ 16:186–194 PMC6057772 30057501

[CR55] Poon IKH, Hulett MD, Parish CR (2009) Molecular mechanisms of late apoptotic/necrotic cell clearance. Cell Death Differ 2010 17(3): 381–397. 10.1038/cdd.2009.195 10.1038/cdd.2009.195 20019744

[CR56] Raghu SV, Kudva AK, Rao S et al (2021) Dietary agents in mitigating chemotherapy-related cognitive impairment (chemobrain or chemofog): first review addressing the benefits, gaps, challenges and ways forward. Food Funct 12:11132–11153. 10.1039/D1FO02391H 34704580 10.1039/d1fo02391h

[CR57] Ramalingayya GV, John J, Gourishetti K et al (2022) Amelioration of Doxorubicin-Induced cognitive impairment by Quercetin in a rat model of breast Cancer. Revista Brasileira De Farmacognosia 1:153–163. 10.1007/s43450-022-00341-y

[CR58] Ribble D, Goldstein NB, Norris DA, Shellman YG (2005) A simple technique for quantifying apoptosis in 96-well plates. BMC Biotechnol. 10.1186/1472-6750-5-12. 5:15885144 10.1186/1472-6750-5-12 PMC1142306

[CR59] Sarder A, Rabbani MG, Chowdhury ASMHK, Sobhani M-E (2015) Molecular Basis of Drug Interactions of Methotrexate, Cyclophosphamide and 5-Fluorouracil as Chemotherapeutic Agents in Cancer. Biomed Res Ther 2:1–11. 10.7603/S40730-015-0005-1

[CR60] Schulze-Osthoff K, Krammer PH, Dröge W (1994) Divergent signalling via APO-1/Fas and the TNF receptor, two homologous molecules involved in physiological cell death. EMBO J 13:4587–4596. 10.1002/J.1460-2075.1994.TB06780.X 7523113 10.1002/j.1460-2075.1994.tb06780.x PMC395391

[CR61] Shackelford RE, Mayhall K, Maxwell NM et al (2013) Nicotinamide phosphoribosyltransferase in malignancy: A review. Genes Cancer 4:447–456. 10.1177/1947601913507576 24386506 10.1177/1947601913507576 PMC3877665

[CR62] Suematsu N, Hosoda M, Fujimori K (2011) Protective effects of Quercetin against hydrogen peroxide-induced apoptosis in human neuronal SH-SY5Y cells. Neurosci Lett 504:223–227. 10.1016/J.NEULET.2011.09.028 21964380 10.1016/j.neulet.2011.09.028

[CR63] Tan X, Yang Y, Xu J et al (2020) Luteolin exerts neuroprotection via modulation of the p62/Keap1/Nrf2 pathway in intracerebral hemorrhage. Front Pharmacol 10:494954. 10.3389/FPHAR.2019.01551/BIBTEX 10.3389/fphar.2019.01551 PMC6985769 32038239

[CR64] Tang H, Wang L, Wang T et al (2023) Recent advances of targeting nicotinamide phosphoribosyltransferase (NAMPT) for cancer drug discovery. Eur J Med Chem 258:115607. 10.1016/J.EJMECH.2023.115607 37413882 10.1016/j.ejmech.2023.115607

[CR65] Vassilopoulos A, Fritz KS, Petersen DR, Gius D (2011) The human Sirtuin family: evolutionary divergences and functions. Hum Genomics 5:485. 10.1186/1479-7364-5-5-485 21807603 10.1186/1479-7364-5-5-485 PMC3230576

[CR66] Venkateshaiah SU, Khan S, Ling W et al (2013) NAMPT/PBEF1 enzymatic activity is indispensable for myeloma cell growth and osteoclast activity. Exp Hematol 41:547–557e2. 10.1016/j.exphem.2013.02.008 23435312 10.1016/j.exphem.2013.02.008 PMC4648259

[CR67] Vichai V, Kirtikara K (2006) Sulforhodamine B colorimetric assay for cytotoxicity screening. Nat Protoc 1(3):1112–1116. 10.1038/nprot.2006.179 17406391 10.1038/nprot.2006.179

[CR68] Wang B, Hasan MK, Alvarado E et al (2011) NAMPT overexpression in prostate cancer and its contribution to tumor cell survival and stress response. Oncogene 30:907–921. 10.1038/onc.2010.468 20956937 10.1038/onc.2010.468

[CR69] Wang G, Han T, Nijhawan D et al (2014) P7C3 neuroprotective chemicals function by activating the Rate-limiting enzyme in NAD salvage. Cell 158:1324. 10.1016/J.CELL.2014.07.040 25215490 10.1016/j.cell.2014.07.040 PMC4163014

[CR70] Wang X, Zhang Q, Bao R et al (2017) Deletion of Nampt in projection neurons of adult mice leads to motor dysfunction, neurodegeneration, and death. Cell Rep 20:2184–2200. 10.1016/j.celrep.2017.08.022 28854367 10.1016/j.celrep.2017.08.022 PMC6021762

[CR71] Wilsbacher JL, Cheng M, Cheng D et al (2017) Discovery and characterisation of novel nonsubstrate and substrate NAMPT inhibitors. Mol Cancer Ther 16:1236–1245. 10.1158/1535-7163.MCT-16-0819/86868/AM/DISCOVERY-AND-CHARACTERIZATION-OF-NOVEL-NON 28468779 10.1158/1535-7163.MCT-16-0819

[CR72] Winocur G, Vardy J, Binns MA et al (2006) The effects of the anticancer drugs, methotrexate and 5-fluorouracil, on cognitive function in mice. Pharmacol Biochem Behav 85:66–75. 10.1016/J.PBB.2006.07.010 16935324 10.1016/j.pbb.2006.07.010

[CR73] Yaku K, Okabe K, Hikosaka K, Nakagawa T (2018) NAD metabolism in Cancer therapeutics. NAD Metabolism Cancer Ther Front Oncol 8:622. 10.3389/fonc.2018.00622 10.3389/fonc.2018.00622 PMC6315198 30631755

[CR74] Yang Q, Kang Z, Zhang J et al (2021) Neuroprotective effects of Isoquercetin: an in vitro and in vivo study citation: neuroprotective effects of Isoquercetin: an in vitro and in vivo study. Cell Journal(Yakhteh) 23:355–365. 10.22074/cellj.2021.7116 10.22074/cellj.2021.7116 PMC8286454 34308580

[CR75] Yao H, Liu M, Wang L et al (2022) Discovery of small-molecule activators of nicotinamide phosphoribosyltransferase (NAMPT) and their preclinical neuroprotective activity. Cell Res 2022 32:6. 10.1038/s41422-022-00651-9 10.1038/s41422-022-00651-9 PMC9160276 35459935

[CR76] Yoo KH, Tang JJ, Rashid MA et al (2021) Nicotinamide mononucleotide prevents cisplatin-induced cognitive impairments. Cancer Res 81:3727–3737. 10.1158/0008-5472.CAN-20-3290 33771896 10.1158/0008-5472.CAN-20-3290 PMC8277702

[CR77] Zak M, Liederer BM, Sampath D et al (2015) Identification of nicotinamide phosphoribosyltransferase (NAMPT) inhibitors with no evidence of CYP3A4 time-dependent Inhibition and improved aqueous solubility. Bioorg Med Chem Lett 25:529–541. 10.1016/J.BMCL.2014.12.026 25556090 10.1016/j.bmcl.2014.12.026

[CR78] Zhai X, Ding Y, Wang Q et al (2016) Rutin acid ameliorates neural apoptosis induced by traumatic brain injury via mitochondrial pathways in mice. Neuroimmunomodulation 23:179–187. 10.1159/000448716 27644033 10.1159/000448716

[CR79] Zhang T, Berrocal JG, Frizzell KM et al (2009) Enzymes in the NAD salvage pathway regulate SIRT1 activity at target gene promoters. 10.1074/jbc.M109.016469 10.1074/jbc.M109.016469 PMC2740465 19478080

[CR80] Zhang M, Lu P, Terada T et al (2021) Quercetin 3,5,7,3′,4′-pentamethyl ether from Kaempferia parviflora directly and effectively activates human SIRT1. Commun Biol 4:1–14. 10.1038/s42003-021-01705-1 33608631 10.1038/s42003-021-01705-1 PMC7896056

[CR81] Zhou SJ, Bi TQ, Qin CX et al (2018) Expression of NAMPT is associated with breast invasive ductal carcinoma development and prognosis. Oncol Lett 15:6648–6654. 10.3892/ol.2018.8164 29725408 10.3892/ol.2018.8164 PMC5920386

