# Supplementary material for: Effects of quercetin and derivatives on NAMPT/Sirtuin-1 metabolic pathway in neuronal cells: an approach to mitigate chemotherapy-induced cognitive impairment
Source: Metab Brain Dis. 2025 Mar 14;40(3):151. doi: 10.1007/s11011-025-01567-0 (PMC11909064; doi:10.1007/s11011-025-01567-0)
Supplement: Supplementary file 1 — Supplementary Material 1 [file 11011_2025_1567_MOESM1_ESM.pdf]

Research Article

Title;

Effects of Quercetin and Derivatives on NAMPT/Sirtuin-1 Metabolic Pathway in Neuronal Cells: An Approach to Mitigate Chemotherapy-Induced Cognitive Impairment

Author Information

Jeena John[a], Subham Das[b], Anu Kunnath[b], Jayesh Mudgal[a], Nandakumar Krishnadas[a*]

[a] Department of Pharmacology, Manipal College of Pharmaceutical Sciences, Manipal Academy of Higher Education, Manipal, Karnataka, India 576104
[b] Department of Pharmaceutical Chemistry, Manipal College of Pharmaceutical Sciences, Manipal Academy of Higher Education, Manipal, Karnataka, India 576104

***Corresponding Author**
Dr. Nandakumar Krishnadas
Professor, Department of Pharmacology
Manipal College of Pharmaceutical Sciences
Manipal Academy of Higher Education
Manipal, Karnataka-576104
Telephone: +91 9008418213
ORCiD: 0000-0001-6653-4660
e-mail: nandakumar.k@manipal.edu, mailnandakumar77@gmail.com

Figures

SIRT1: Molecular Dynamics

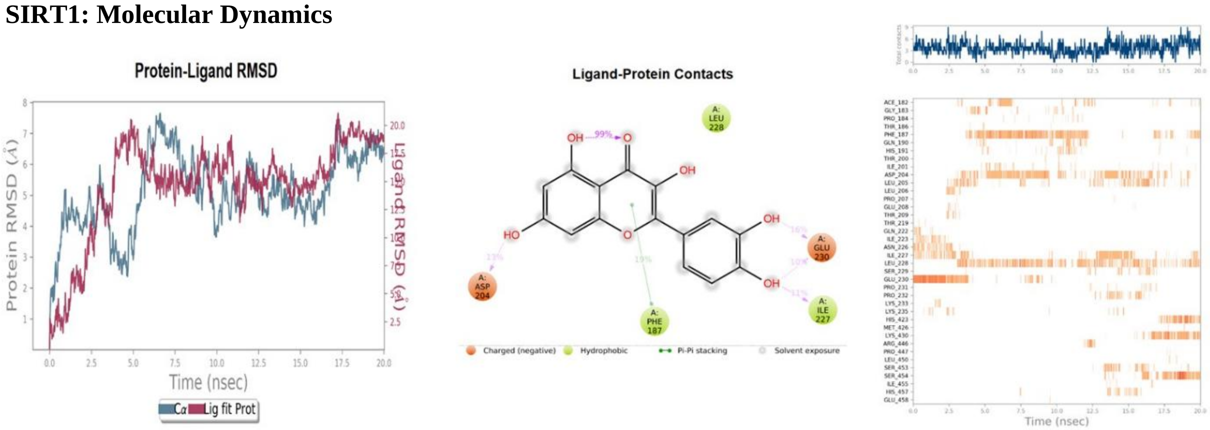

Fig. S1 RMSD Plot, ligand-protein contacts of Quercetin in 20ns time frame in SIRT1 protein

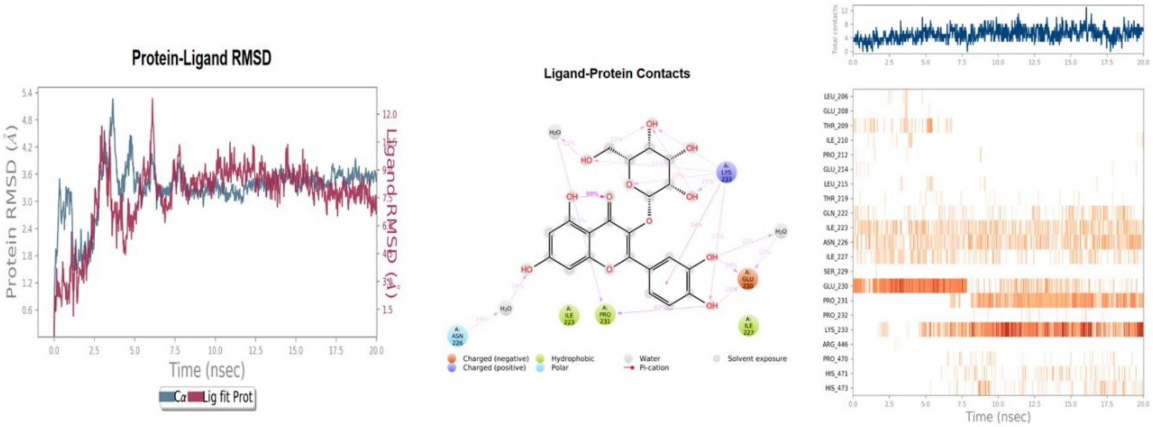

Fig. S2 RMSD Plot, ligand-protein contacts of Isoquercetin in 20ns time frame in SIRT1 protein

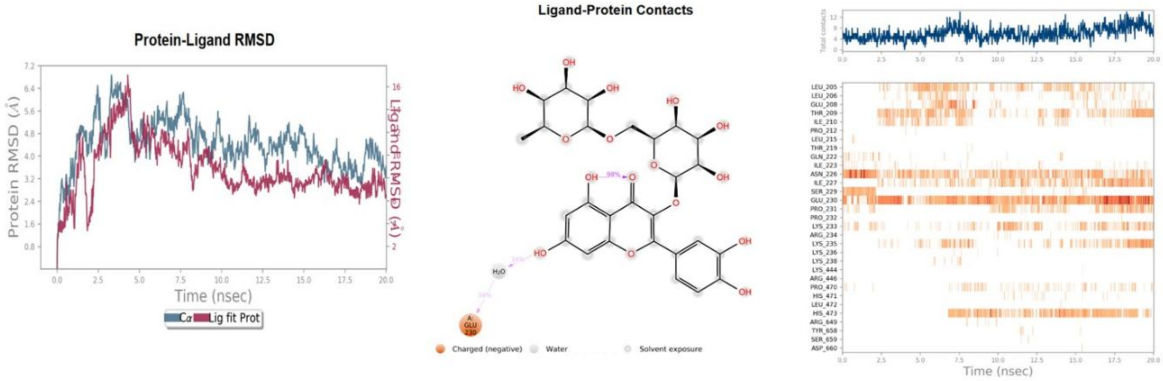

Fig. S3 RMSD Plot, ligand-protein contacts of Rutin in 20ns time frame in SIRT1 protein

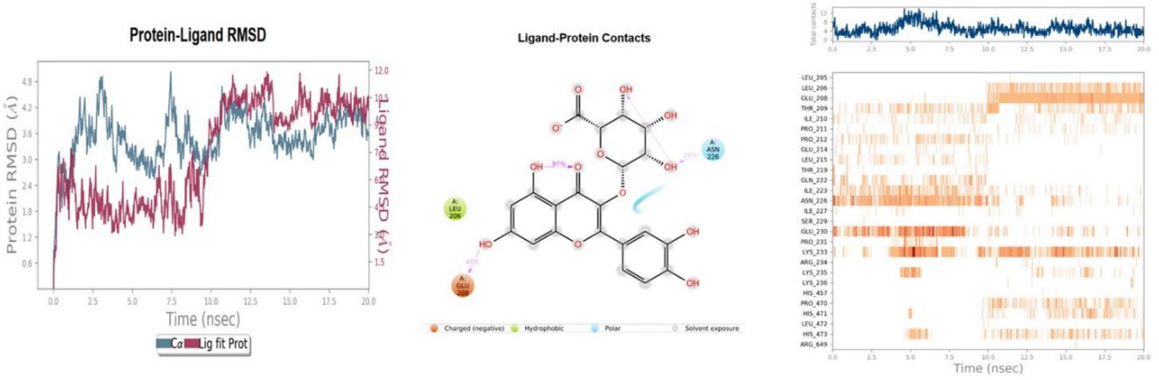

Fig. S4 RMSD Plot, ligand-protein contacts of Miquelianin in 20ns time frame in SIRT1 protein

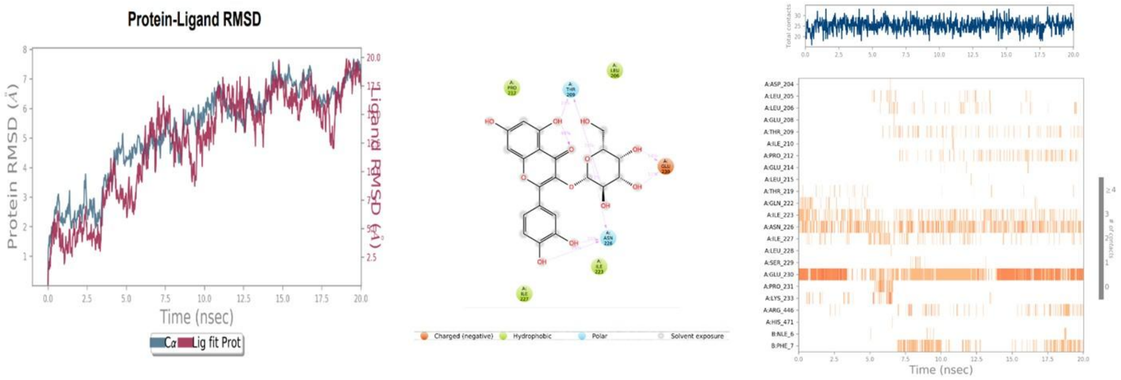

Fig. S5 RMSD Plot, ligand-protein contacts of Hyperoside in 20ns time frame in SIRT1 protein

NAMPT: Molecular Dynamics

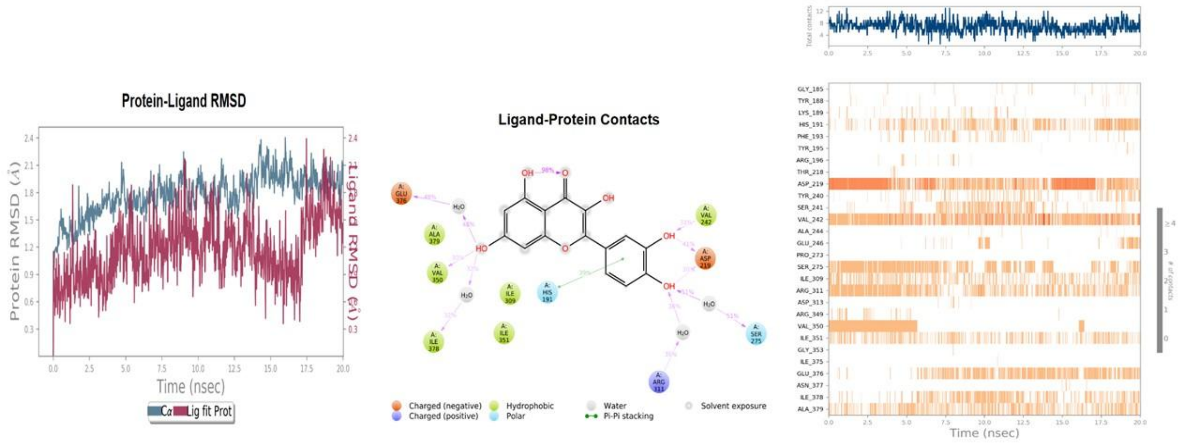

Fig. S6 RMSD Plot, ligand-protein contacts of Quercetin in 20ns time frame in NAMPT protein

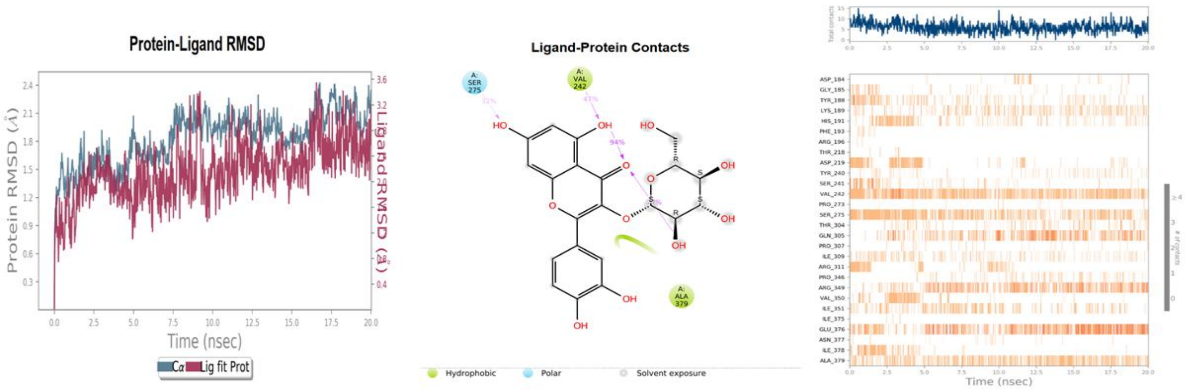

Fig. S7 RMSD Plot, ligand-protein contacts of Isoquercetin in 20ns time frame in NAMPT protein

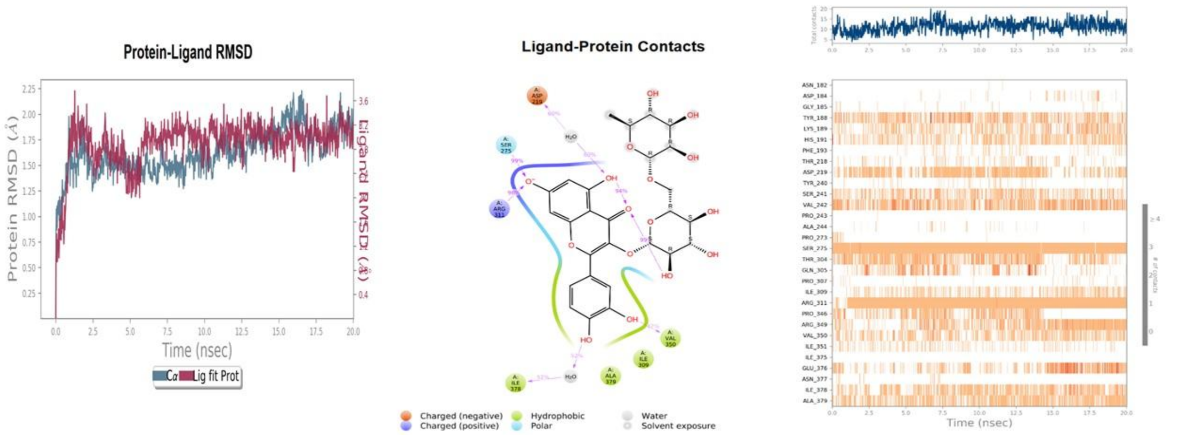

Fig.S8 RMSD Plot, ligand-protein contacts of Rutin in 20ns time frame in NAMPT protein

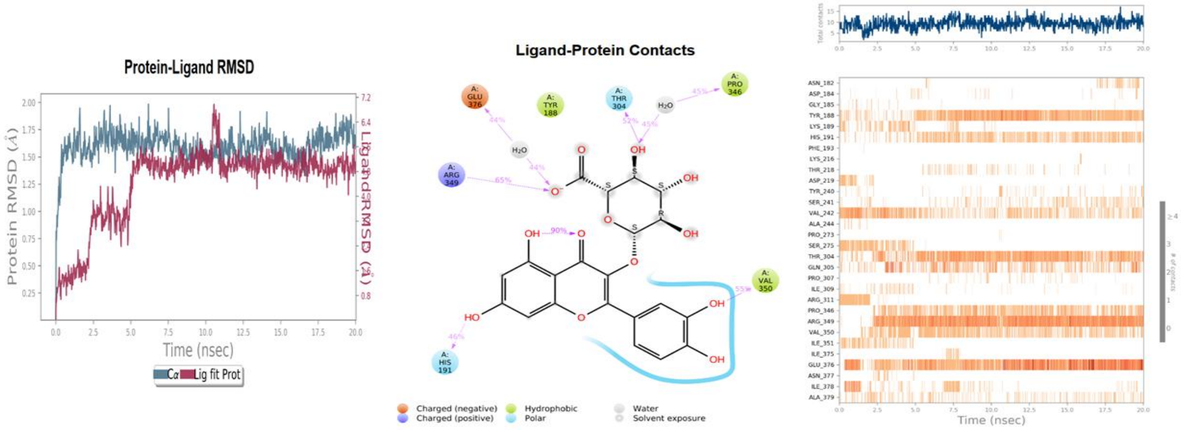

Fig. S9 RMSD Plot, ligand-protein contacts of Miquelianin in 20ns time frame in NAMPT protein

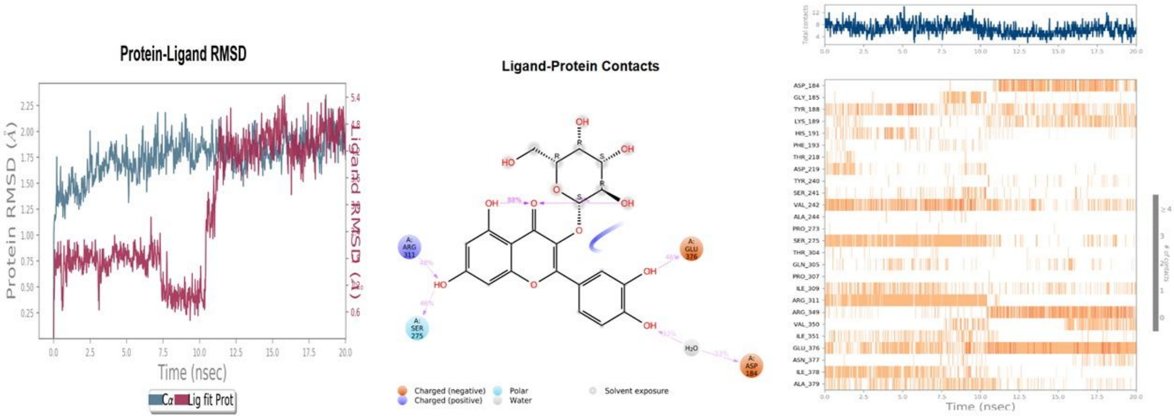

Fig. S10 RMSD Plot, ligand-protein contacts of Hyperoside in 20ns time frame in NAMPT protein

Tables

Ligand	Hydrogen bonding	Hydrophobic Interactions	Hydrophilic Interactions	2-D Interaction diagram
**Co-ligand (4TQ)**	GLU 230 (negative charge)	LEU 215, PRO 212, PRO 211, LEU 206, ILE 223, ILE 227, PRO 230	THR 209, ASN 226, SER 229	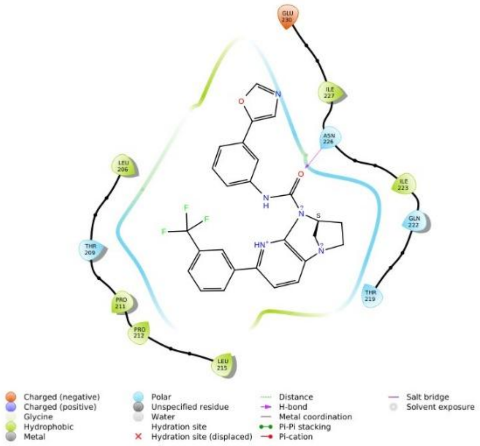

**Quercetin**	2GLU 230, ASN 226	ILE 223, ILE 227	THR 219, ASN 226, GLN 222	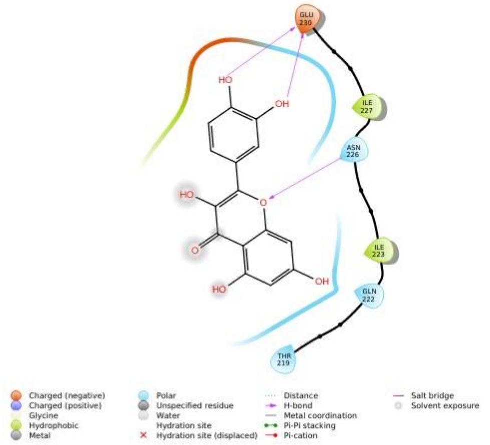
**Rutin**	4 GLU 230, ASN 226	PRO 231, ILE 223, ILE 227	THR 219, ASN 226, GLN 222 Other: LYS 233(positive charge)	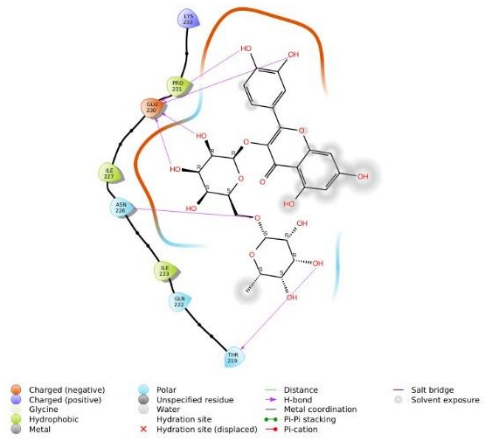
**Hyperoside**	2GLU 230, ASN 226	LEU 215, ILE 223, ILE 227, LEU 206	THR 209, THR 219, ASN 226	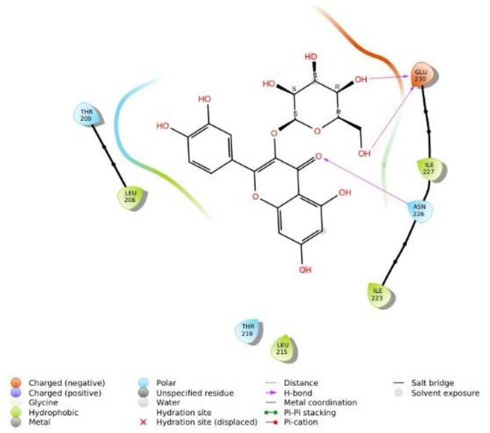

**Isorhamnetin**	ASN 226	LEU 215, ILE 223, ILE 227, LEU 206, PRO 212	THR 219, ASN 226 Other: GLU 230(negative charge)	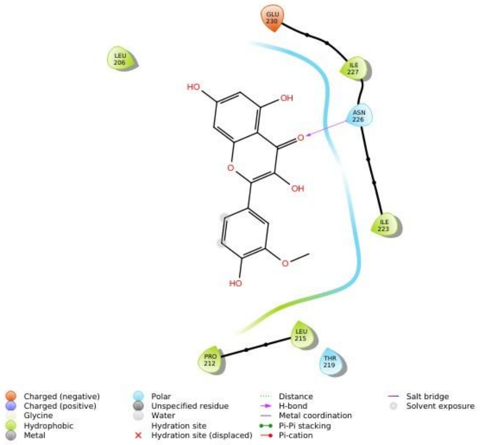
**Miquelianin**	2GLU 230, ASN 226	ILE 223, ILE 227, LEU 206	THR 209, THR 219, ASN 226, GLN 222	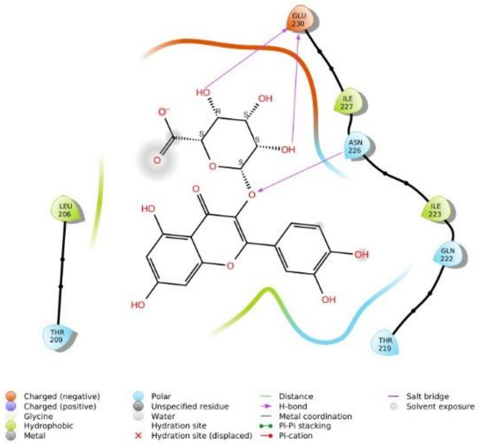
**Catechin**	2GLU 230, ASN 226, GLN 222, THR 219	ILE 223, ILE 227, LEU 215	THR 219, ASN 226, GLN 222	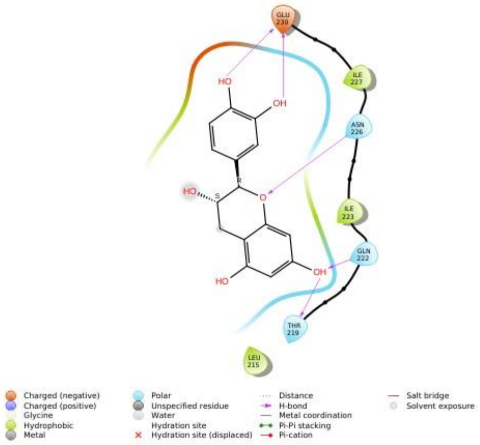

**Isoquercetin**	2GLU 230, ASN 226	ILE 223, ILE 227, LEU 206, PRO 211, PRO 212, LEU 215	THR 209, THR 219, ASN 226	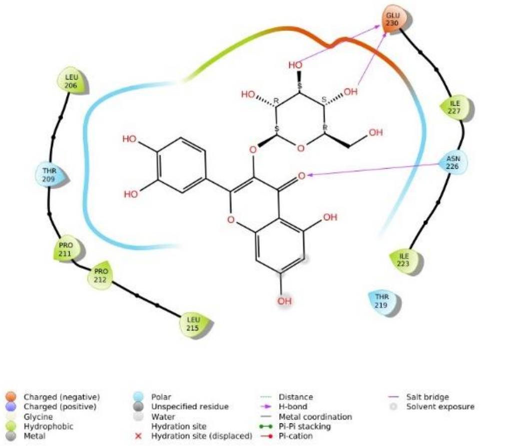
**Luteolin**	2GLU 230, ASN 226	ILE 223, ILE 227	THR 209, THR 219, ASN 226	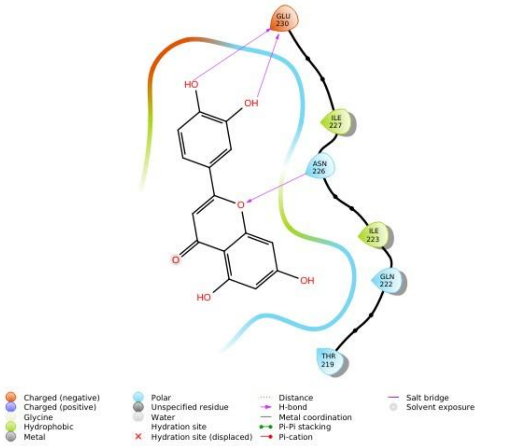
**Myricetin**	GLN 222, ASN 226	ILE 223, ILE 227, LEU 206, PRO 212, LEU 215	THR 209, THR 219, ASN 226, GLN 222	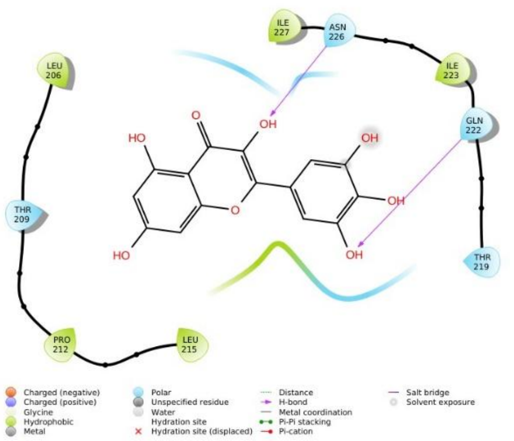

**Morin**	GLU 230, ASN 226	ILE 223, ILE 227, LEU 215	GLN 222, THR 219, ASN 226	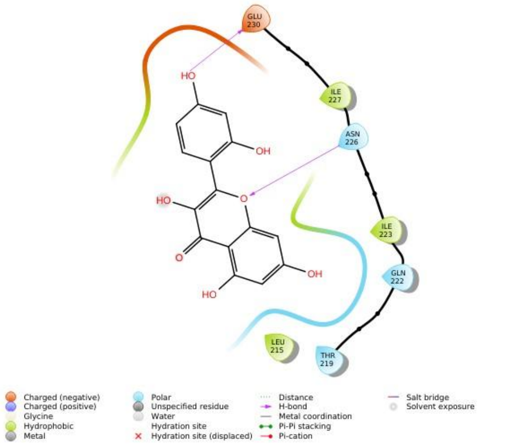
**Apigenin**	GLU 230, ASN 226	ILE 223, ILE 227	GLN 222, THR 219, ASN 226	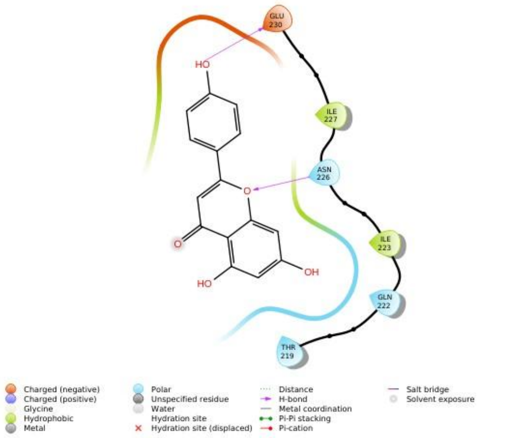
**Taxifolin**	2GLU 230, ASN 226	ILE 223, ILE 227	THR 219, GLN 222, ASN 226	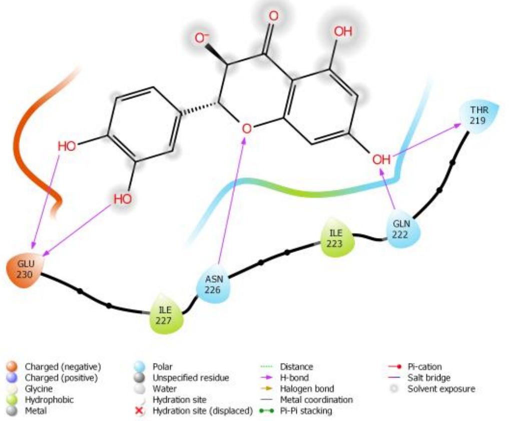

Table S1: 2D interaction diagrams including interactions with SIRT1 protein

Ligand	Hydrogen bonding	Hydrophilic Interactions	Hydrophobic Interactions	2-D Interaction diagram
**Co-ligand (NAT)**	ASP 219, SER 275	SER 275, SER 241, HIE 191	PRO 273, ILE 351, ILE 309, PRO 307, ALA 244, VAL 242, PHE 193, ALA 379, TYR 188, TYR 240	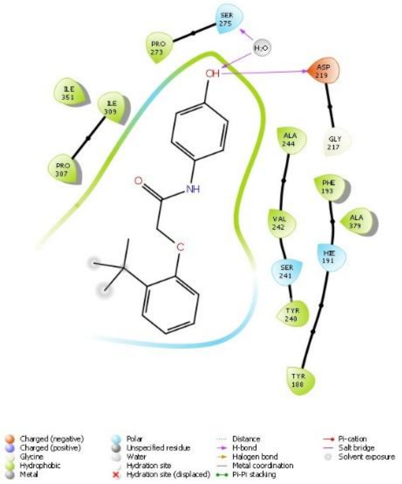
**Quercetin**	ASP 219, SER 275, VAL 242, VAL 350, LYN 189	SER 275, SER 241, HIE 191, ASN 377	ILE 351, ILE 309, ALA 244, VAL 242, PHE 193, ALA 379, TYR 188, VAL 350, ILE 378	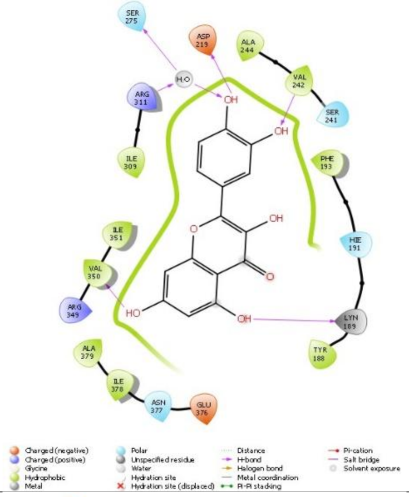
**Rutin**	LYN 189, ASP 219, VAL 242, ASP 184	SER 241, THR 218, HIE 191	ILE 378, ALA 379, PRO 307, ILE 309, ILE 351, VAL 350, TYR 188, PHE 193, VAL 242, ALA 244, TYR 240	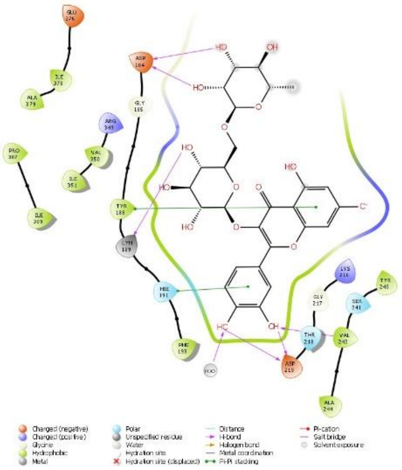

**Hyperoside**	ASP 219, LYN 189, SER 275, VAL 242, VAL 350	SER 275, SER 241, HIE 191, ASN 377	ILE 351, ALA 379, PRO 273, PRO 307, ILE 309, VAL 242, ALA 244, TYR 188, ILE 378, PHE 193, TYR 240, VAL 350	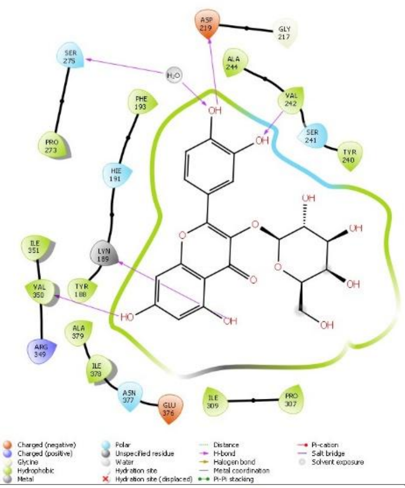
**Isorhamnetin**	ASP 219	SER 275, SER 241, HIE 191, ASN 377	ILE 351, ALA 379, ILE 309, VAL 242, ALA 244, TYR 188, PHE 193, VAL 350, ILE 378	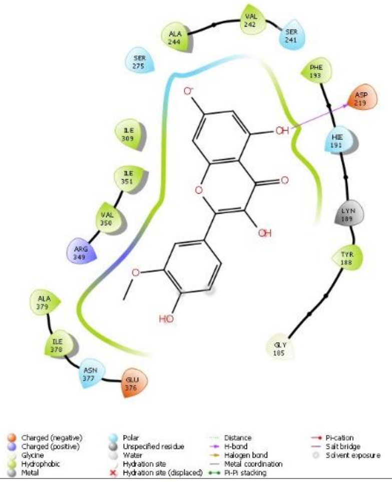
**Miquelianin**	ASP 219, LYN 189, VAL 242, SER 275, GLN 305	SER 275, SER 241, HIE 191, GLN 305	PRO 273, ILE 309, PRO 307, TYR 240, VAL 242, ALA 244, PHE 193, ILE 351, VAL 350, TYR 188, ALA 379, ILE 378	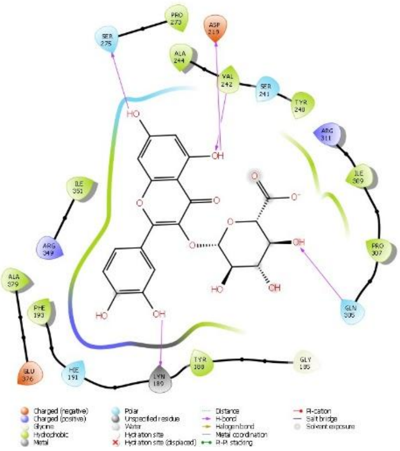

**Isoquercetin**	ASP 219, LYN 189, SER 275. ARG 311, VAL 242	SER 275, SER 241, HIE 191	PRO 273, ILE 309, PRO 307, TYR 240, VAL 242, ALA 244, PHE 193, ILE 351, VAL 350, TYR 188, ALA 379, ILE 378	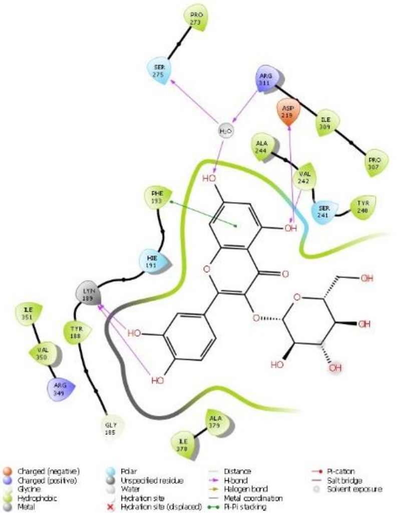
**Luteolin**	ASP 219, SER 275, VAL 242,	SER 241, HIE 191, SER 275, ASN 377	ALA 244, VAL 242, PHE 193, TYR 188, ILE 351, VAL 350, ILE 309, ILE 378, ALA 379, ILE 351	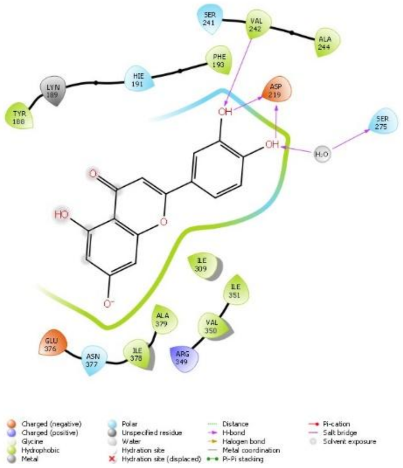
**Myricetin**	ASP 219, SER 275, VAL 242,	SER 241, HIE 191, SER 275	ALA 244, VAL 242, PHE 193, TYR 188, VAL 350, ILE 309, ILE 378, ALA 379, ILE 351	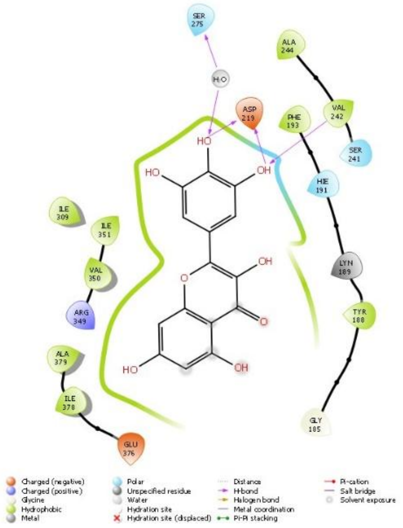

**Morin**	ASP 219, SER 275	SER 241, HIE 191, SER 275	ALA 244, VAL 242, TYR 188, ILE 309, ALA 379, ILE 351, ***Pi-Pi*****:** PHE 193	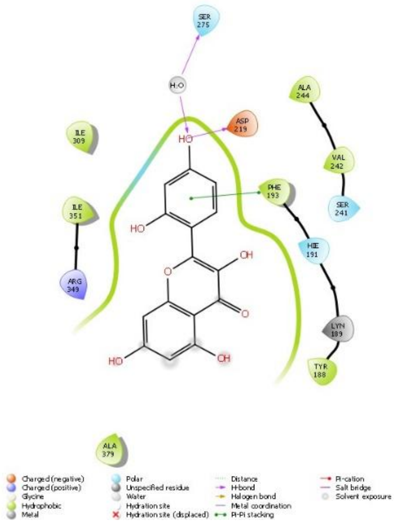
**Apigenin**	ASP 219, SER 275	SER 241, HIE 191, SER 275	ALA 244, VAL 242, PHE 193, TYR 188, VAL 350, ILE 309, ALA 379, ILE 351	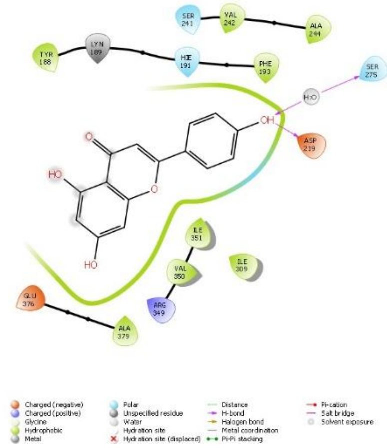
**Taxifolin**	ASP 219, SER 275, VAL 242, HIE 191	SER 241, HIE 191, SER 275	ALA 244, VAL 242, PHE 193, TYR 188, VAL 350, ILE 309, ALA 379, ILE 351, ILE 378	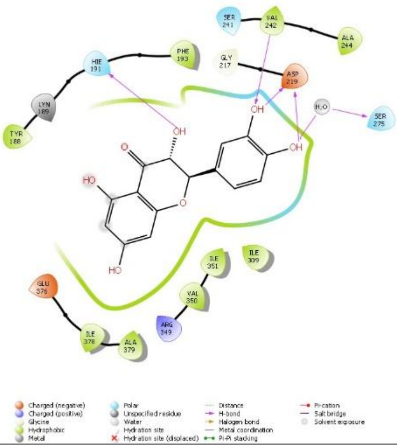

Table S2: 2D interaction diagrams including interactions with NAMPT protein

Treatment	Concentration (μM)	Percentage of Cell viability in SH-SY5Y Cells	
		**Undifferentiated**	**Differentiated**
**Normal Control**	-	100	100
**Methotrexate+5-Fluorouracil**	4.179	49.53±2.363####	55.46±2.664####
**Quercetin**	0.00096	60.83±1.092****	67.26±0.8592****
	0.0048	63.14±1.429****	72.02±1.375****
	0.24	63.79±1.388****	73.66±0.601****
	0.12	64.39±1.301****	74.70±0.343****
	0.6	62.76±0.889****	77.53±1.289****
	3	60.96±1.303****	70.98±1.632****
	15	59.76±0.480****	69.20±0.773****

Table S3: Effect of 2 h pretreatment of quercetin on cell viability on MF-induced toxicity in SH-SY5Y cells. **A)** Undifferentiated SH-SY5Y cells **B)** Differentiated SH-SY5Y cells. Data represents mean±SEM analysed by One-way ANOVA followed by Dunnet's post-hoc test. ####p<0.0001 compared to normal control, ****p<0.0001 compared to MF control.

Treatment	Concentration (μM)	Percentage of Cell viability in SH-SY5Y Cells	
		Undifferentiated	Differentiated
**Normal Control**	-	100	100
**Methotrexate+5-Fluorouracil**	4.179	49.53±2.363####	55.46±2.664####
**Rutin**	0.128	60.24±1.182****	73.21±1.203****
	0.64	59.76±0.594****	74.23±0.839****
	3.2	64.13±2.016****	74.70±0.343****
	16	64.13±0.926****	73.07±1.976****
	80	58.14±1.157****	76.64±0.601****
	400	61.30±1.049****	66.67±1.718****
	2000	56.77±0.816****	55.51±1.289

Table S4: Effect of 2 h pretreatment of rutin on cell viability on MF-induced toxicity in SH-SY5Y cells. **A)** Undifferentiated SH-SY5Y cells **B)** Differentiated SH-SY5Y cells. Data represents mean±SEM analysed by One-way ANOVA followed by Dunnet's post-hoc test. ####p<0.0001 compared to normal control. ****p<0.0001 compared to MF control.

**Treatment**	**Concentration (µM)**	**Percentage of Cell viability in SH-SY5Y Cells**	
		Undifferentiated	Differentiated
**Normal Control**	-	100	100
**Methotrexate+5-Fluorouracil**	4.192	49.53±2.363####	55.46±2.664####
**Isoquercetin**	0.128	60.88±0.546****	80.51±1.976****
	0.64	58.10±2.875***	72.77±1.976****
	3.2	63.10±2.605****	82.29±0.773****
	16	62.12±2.919****	86.61±1.031****
	80	57.50±3.401***	86.76±0.601****
	400	52.19±0.926	70.54±0.171****
	2000	43.34±1.112	55.51±0.257

Table S5: Effect of 2 h pretreatment of isoquercetin on cell viability on MF-induced toxicity in SH-SY5Y cells. **A)** Undifferentiated SH-SY5Y cells **B)** Differentiated SH-SY5Y cells. Data represents mean±SEM analysed by One-way ANOVA followed by Dunnet's post-hoc test. ####p<0.0001 compared to normal control. ***p<0.001, ****p<0.0001 compared to MF control.

**Treatment**	**Concentration (µM)**	**Neurite Length**
**Normal Control**	-	85.38±6.717
**Methotrexate+5-Fluorouracil**	4.179	41.77±4.715####
**Quercetin**	0.024	81.57±3.805****
	0.12	81.63±3.428****
	0.6	82.53±4.624****
	3	80.44±4.203****
	15	76.74±5.916***
**Rutin**	3.2	79.01±4.500****

	16	79.72±4.537****
	80	83.83±5.148****
	400	64.85±3.451*
**Isoquercetin**	3.2	75.15±3.798****
	16	81.07±3.769****
	80	85.35±4.738****
	400	73.11±4.606****

Table S6: Effect of 2h pretreatment of QRT, RUT and IQRT concentrations on the neurite length on MF-induced toxicity in differentiated SH-SY5Y cells after 48h of incubation. Data represents mean±SEM of three trials analysed by One-way ANOVA followed by Dunnet's post-hoc test. ####p<0.0001 compared to normal control group, *p<0.05,***p<0.001, ****p<0.0001 compared to MF control group
